# Re-analyses of “Algal” Genes Suggest a Complex Evolutionary History of Oomycetes

**DOI:** 10.3389/fpls.2017.01540

**Published:** 2017-09-06

**Authors:** Qia Wang, Hang Sun, Jinling Huang

**Affiliations:** ^1^Key Laboratory for Plant Diversity and Biogeography of East Asia, Kunming Institute of Botany, Chinese Academy of Sciences Kunming, China; ^2^University of Chinese Academy of Sciences Beijing, China; ^3^State Key Laboratory of Cotton Biology, Institute of Plant Stress Biology, Henan University Kaifeng, China; ^4^Department of Biology, East Carolina University, Greenville NC, United States

**Keywords:** plastid evolution, stramenopiles, endosymbiosis, horizontal gene transfer, eukaryotic evolution

## Abstract

The spread of photosynthesis is one of the most important but constantly debated topics in eukaryotic evolution. Various hypotheses have been proposed to explain the plastid distribution in extant eukaryotes. Notably, the chromalveolate hypothesis suggested that multiple eukaryotic lineages were derived from a photosynthetic ancestor that had a red algal endosymbiont. As such, genes of plastid/algal origin in aplastidic chromalveolates, such as oomycetes, were considered to be important supporting evidence. Although the chromalveolate hypothesis has been seriously challenged, some of its supporting evidence has not been carefully investigated. In this study, we re-evaluate the “algal” genes from oomycetes with a larger sampling and careful phylogenetic analyses. Our data provide no conclusive support for a common photosynthetic ancestry of stramenopiles, but show that the initial estimate of “algal” genes in oomycetes was drastically inflated due to limited genome data available then for certain eukaryotic lineages. These findings also suggest that the evolutionary histories of these “algal” genes might be attributed to complex scenarios such as differential gene loss, serial endosymbioses, or horizontal gene transfer.

## Introduction

How photosynthesis evolved in eukaryotes has been a subject of tremendous scientific interest. Oxygenic photosynthesis was first invented by cyanobacteria (Gould et al., 2008). During the early evolution of eukaryotes, a cyanobacterial cell was engulfed by a heterotrophic eukaryote (Margulis, 1970; Martin and Kowallik, 1999; McFadden, 2001; Palmer, 2003), spawning the origin of primary plastids and Plantae (also called Archaeplastida, including green plants, red algae, and glaucophytes) (Cavalier-Smith, 1981; Delwiche and Palmer, 1997; Gould et al., 2008). This process was accompanied by massive cyanobacterial gene loss and transfer to the host nucleus. Subsequently, the photosynthetic capacity was spread to multiple other eukaryotic lineages through higher-order endosymbioses (secondary, tertiary, or quaternary) (Delwiche, 1999), that is, these eukaryotes acquired plastids by engulfing another photosynthetic eukaryote instead of a cyanobacterial cell. Although it is clear that the spread of photosynthetic capacity in eukaryotic lineages represents a history of reticulate evolution involving multiple endosymbiotic events, the exact number and the nature of historical endosymbioses remain controversial.

Among eukaryotic lineages involved in higher-order endosymbioses, it was generally accepted that plastids of euglenids and chlorarachniophytes are derived from green algal endosymbionts (Gibbs, 1978; Ludwig and Gibbs, 1989; Van de Peer et al., 1996), whereas plastids of cryptophytes, alveolates, stramenopiles and haptophytes (CASH lineages) are from red algal endosymbionts (Cavalier-Smith, 1995; Cavalier-Smith et al., 1996; Palmer and Delwiche, 1998; Delwiche, 1999). For a long period of time, plastid gains through endosymbiotic events were considered to be extremely rare and plastid losses, on the other hand, were thought to be relatively common. Such a belief also formed the foundation of the Cabozoa hypothesis and the chromalveolate hypothesis (Cavalier-Smith, 1999; Cavalier-Smith and Chao, 2003). The Cabozoa hypothesis argued that plastids of euglenids and chlorarachniophytes could be traced back to a common secondary endosymbiotic event involving a green alga (Cavalier-Smith, 1999; Cavalier-Smith and Chao, 2003). Similarly, the chromalveolate hypothesis proposed that plastids in CASH lineages were vertically derived from a common ancestor that engulfed a red algal endosymbiont and, as such, aplastidic organisms in these lineages were interpreted as resulting from secondary plastid losses (Cavalier-Smith, 1999). However, multiple lines of evidence (Baldauf et al., 2000; Archibald et al., 2003; Leander, 2004; Gilson et al., 2006), including the complete chloroplast genome of chlorarachniophyte *Bigelowiella natans* (Rogers et al., 2007), rejected the Cabozoa hypothesis. Thus far, it is commonly believed that the plastids of euglenids and chlorarachniophytes were acquired from two independent green algal endosymbiotic events (Gould et al., 2008; Keeling, 2013). The chromalveolate hypothesis has long been under debate and now in jeopardy in face of recent data (Bodył, 2005; Burki et al., 2008, 2012b, 2016; Kim and Graham, 2008; Yoon et al., 2008; Bodył et al., 2009). Several other hypotheses have been proposed, each of which is supported by different lines of evidence (Bodył and Moszczyński, 2006; Sanchez-Puerta and Delwiche, 2008; Okamoto et al., 2009; Stiller et al., 2014). Therefore, how plastids evolved in red plastid lineages remains unsettled.

Stramenopiles (also known as heterokonts), as a major eukaryotic clade, include a wide variety of organisms (Cavalier-Smith, 1986; Patterson, 1989). This lineage contains not only many important algae, such as diatoms that are a major producer of oxygen and consumer of carbon dioxide in marine ecosystems, but also a significant fraction of aplastidic or heterotrophic organisms, including pathogens like *Phytophthora infestans*, the causative agent of potato late blight that triggered the Great Irish Famine in the 1840s. Whether these diverse organisms originated from a common photosynthetic ancestor is crucial for understanding the evolution of stramenopiles as well as eukaryotes in general. This in turn led to many studies on the existence of potential historical plastids in heterotrophic stramenopiles (Keeling, 2013).

Oomycetes are fungus-like eukaryotic microorganisms that often have a saprophytic or pathogenic lifestyle. Oomycetes were once placed within fungi in earlier classification systems, but are now widely considered as part of stramenopiles (Baldauf et al., 2000; Yoon et al., 2002). Although there are different views about the phylogenetic relationships within stramenopiles (Brown and Sorhannus, 2009; Riisberg et al., 2009; Yang et al., 2012; Cavalier-Smith and Scoble, 2013; Ševčíková et al., 2015), the most recent phylogenomic analyses suggest that oomycetes form a clade closely related to ochrophytes, a monophyletic group of photosynthetic stramenopiles (Derelle et al., 2016). Unlike ochrophytes, oomycetes do not contain plastids (Tyler et al., 2006; Derelle et al., 2016), not even vestigial ones like those in apicomplexan parasites (called apicoplast) (Maréchal and Cesbron-Delauw, 2001). If all stramenopiles are derived from a single photosynthetic ancestor, plastids would have been lost in oomycetes.

In 2006, draft genome sequences of two oomycete species, *Phytophthora sojae* and *P. ramorum*, were published (Tyler et al., 2006). In this study, 855 genes of putatively algal origin (“algal” genes hereafter) were identified based on their unusually high similarities to sequences from algae and/or cyanobacteria, 30 of which were considered the most likely cases after detailed phylogenetic analyses. These “algal” genes were interpreted as the relic from a red algal endosymbiont (plastid) and subsequent endosymbiotic gene transfer (EGT) or endosymbiotic gene replacement (EGR). As key evidence for historical plastids in oomycetes, these “algal” genes were further used to support the hypothesis that all stramenopiles were derived from a photosynthetic ancestor. Such evidence, however, has been called into question by a more recent statistical genomic analysis that found no unusual contribution from a red algal endosymbiont to *Phytophthora* genomes (Stiller et al., 2009).

Like in many earlier studies on gene transfer, insufficient taxonomic sampling was a potential caveat for the identification of “algal” genes in oomycetes. This is evidenced by the fact that, although the identified “algal” genes were interpreted as derived from a red algal endosymbiont, their sequences, on the other hand, were often found to be closely related to green algal homologs, presumably due to the lack of sufficient sequence data from red algae. As more genome sequence data from various major eukaryotic lineages become available in recent years, we now revisit the “algal” genes identified in oomycete genomes. Our goal is to provide a better understanding of the nature of these genes and the potential interactions of oomycetes/stramenopiles with other organisms, particularly primary photosynthetic eukaryotes.

## Materials and Methods

### Data Sources

In the original *Phytophthora* genome analyses, 855 genes were considered to be of algal or cyanobacterial origin, and 30 most likely candidates were subject to further detailed analyses (Tyler et al., 2006). We downloaded the protein sequences of these 30 genes of *Phytophthora ramorum* from http://www.jgi.doe.gov/Pramorum, and used them as queries to search the National Center for Biotechnology Information (NCBI) non-redundant (nr) protein sequences database (*E*-value cutoff 1*e*-7). Additional searches were also performed against over 650 transcriptomes in the Marine Microbial Eukaryote Transcriptome Sequencing Project (MMETSP) (Keeling et al., 2014), the fungal genome database at the Joint Genome Institute^1^, and our internal customized database (Supplementary Table 1). Particularly, a total of six red algal genomes of five genera were used to search for *P. ramorum* gene homologs, including *Cyanidioschyzon merolae*, *Porphyridium purpureum*, *Chondrus crispus*, *Galdieria sulphuraria*, *G. phlegrea*, and *Pyropia yezoensis*. Complete genome sequence data of multiple photosynthetic stramenopiles (including *Aureococcus anophagefferens*, *Ectocarpus siliculosus*, *Fragilariopsis cylindrus*, *Nannochloropsis gaditana*, *Phaeodactylum tricornutum*, *Saccharina japonica*, and *Thalassiosira pseudonana*) were also searched in our analyses.

### Re-analyses of BLAST Results

In the original *Phytophthora* genome paper (Tyler et al., 2006), the search of “algal” genes was based on significant matches to sequences from Plantae and cyanobacteria (that is, these sequence matches had the highest bit scores and the lowest *E*-values outside the stramenopiles). The *Phytophthora* “algal” genes identified from the BLAST search were shared by other stramenopiles (or chromalveolates), and they had stronger BLAST matches to homologous genes of red algae and/or cyanobacteria than to sequences from archaea, opisthokonts or non-cyanobacterial bacteria. Additionally, a complementary approach based on Smith–Waterman alignment was also used to identify candidates with significantly higher similarities to red algal or green plant homologs than to those from opisthokonts or amoebozoans. Because *Cyanidioschyzon merolae*, which also happens to have a streamlined genome, was the only red alga whose complete nuclear genome sequence was then available, the matches to green plant homologs were included and interpreted as resulting from the lack of sufficient red algal genome data (Tyler et al., 2006).

In the current study, BLAST search was performed against NCBI nr, MMETSP and our internal customized databases for each of the 30 most likely “algal” genes, followed by re-analyses of its phylogenetic distribution and gene structure. Following the criteria used by the *Phytophthora* genome paper (Tyler et al., 2006), we compared the best BLAST matches (represented by the highest bit scores) between homologs from red algae, cyanobacteria, photosynthetic stramenopiles and those from archaea, opisthokonts, amoebozoans, and non-cyanobacterial bacteria. Because more red algal genomes and transcriptomic data were included in our analyses, the matches to green plant homologs were no longer included and used as proxy for red algal homologs.

### Phylogenetic Analyses

For each of the 30 “algal” genes identified in *Phytophthora* genome sequencing project, we performed further phylogenetic analyses. In order to attain a broad and balanced sampling, we selected protein sequences from representative groups of three domains of life (eukaryotes, bacteria, and archaea). The same sampling strategy was also used to ensure sufficient coverage of representative taxa within each major eukaryotic group. This was done using a Perl script followed by manual inspection and additional sequence sampling if needed. Particular attention was paid to groups under-sampled in the previous analyses, such as chromalveolates and other protists. Multiple alignments of sampled sequences were performed using MUSCLE (Edgar, 2004), followed by careful manual inspection of alignment quality, gene structure, shared insertions/deletions (indels), and conserved amino acid residues. Gaps, ambiguously aligned sites, and sequences whose real identity could not be confirmed were removed from alignments. Phylogenetic analyses were performed with maximum likelihood method using PhyML 3.1 (Guindon et al., 2010) and distance method using neighbor of PHYLIP-3.695 (Felsenstein, 2013). The optimal model of protein substitution and rate heterogeneity were chosen based on the result of ModelGenerator (Keane et al., 2006). Bootstrap analyses were performed using 1,000 replicates.

## Results

### The Identity of “Algal” Genes in Oomycetes

If the previously identified “algal” genes in *Phytophthora* are indeed derived from a red algal endosymbiont acquired by the ancestor of stramenopiles, their homologs might also be found in photosynthetic stramenopiles. Given their presumably red algal nature, these stramenopile sequences theoretically should have a closer relationship to homologs from red algae (or red algae and other photosynthetic eukaryotes plus cyanobacteria) than to those from other organisms (e.g., opisthokonts, amoebozoans, non-cyanobacterial bacteria, and archaea). Our BLAST results with a larger taxonomic sampling showed that, for all of the 30 most likely “algal” genes previously identified in *Phytophthora*, only 10 of them (about 33%) were more similar to sequences of red algae, photosynthetic eukaryotes and/or cyanobacteria (**Table 1**); these 10 genes were also the viable candidate genes of red algal origin.

**Table 1:** BLAST search results of 30 most likely “algal” genes in *Phytophthora*.

			Best BLASTp match (represented by bit scores)						
*P. ramorum*	Putative gene	Target	Cyanobacteria	Red algae	Photosynthetic	Opisthokonts/	Non-cyanobacterial	Archaea	Figure
Gene ID	product				stramenopiles	amoebozoans	bacteria		
72019	Cobalamin-independent methionine synthase		805	975	861	759	808	555	**Figure 1**
54177	Prolyl oligopeptidase II		633	822	733	493	630	214	Supplementary Figure 1
75281	2-Isopropylmalate synthase	^∗^	593	543	545	**618**	**600**	528	**Figure 6**
54068	Threonine ammonia-lyase	^∗^	482	469	571	**503**	**508**	218	Supplementary Figure 2
79142	Anthranilate synthase	^∗^	580	498	543	474	562	258	Supplementary Figure 3
38584	NCAIR mutase	^∗^	234	none	224	126	218	193	**Figure 3**
74880	3′-phosphoadenosine 5′-phosphosulfate reductase		268	217	281	135	256	120	**Figure 7**
51635	Uroporphyrinogen-III methyltransferase	^∗^	217	174	161	199	213	204	Supplementary Figure 4
95818	tRNA (guanine-N(7)-)- methyltransferase-like	^∗^	176	148	149	91	162	none	Supplementary Figure 5
80275	Phosphatidate cytidylyltransferase		108	87	85.9	102	**130**	none	Supplementary Figure 6
87801	Ketol-acid reductoisomerase	^∗^	599	639	665	190	**640**	212	**Figure 5**
80380	Phosphoserine aminotransferase	^∗^	393	407	436	405	**417**	372	Supplementary Figure 7
72085	Asparaginyl tRNA synthetase	^∗^	478	482	474	472	**486**	256	Supplementary Figure 8
75838	SAICAR synthetase	^∗^	215	383	427	172	**388**	256	Supplementary Figure 9
72293	Glucokinase		255	278	328	265	237	127	**Figure 4**
75742	Histidinol-phosphate aminotransferase		99	332	491	**426**	116	86.7	Supplementary Figure 10
78949	Zinc carboxypeptidase A		none	259	379	71	75	64	Supplementary Figure 11
79657	cAMP-binding mitochondrial solute carrier		60.8	313	131	157	107	81	**Figure 2**
45002	Enoyl-(acyl-carrier-protein) reductase	^∗^	204	209	186	183	**223**	144	Supplementary Figure 12
77863	Sulfur transferase + methyl transferase fusion		166	296	288	186	176	127	Supplementary Figure 13
86425	Methylthioadenosine phosphorylase		78	167	none	**168**	127	142	Supplementary Figure 14
54215	Ribonuclease HII		160	185	230	154	**199**	81.3	Supplementary Figure 15
71442	Nitrate reductase		141	717	703	625	224	115	Supplementary Figure 16
71783	6-phosphogluconate dehydrogenase		549	545	799	**559**	538	538	Supplementary Figure 17
83828	Aspartate kinase/homoserine dehydrogenase		180	222	214	157	**233**	71	Supplementary Figure 18
73217	Galactonolactone dehydrogenase		158	418	417	409	147	140	Supplementary Figure 19
85610	Cobalamin synthesis protein		314	319	379	**369**	**325**	167	Supplementary Figure 20
78464	tRNA dihydrouridine synthase		236	240	332	187	**253**	88	Supplementary Figure 21
72218	Folate-biopterin transporter		253	239	331	65	211	none	Supplementary Figure 22
82990	Prephenate dehydratase family	^∗^	95.9	127	199	**157**	**160**	**148**	Supplementary Figure 23

*Phytophthora ramorum gene IDs are from the JGI database. Genes whose protein products are presumably localized in mitochondria in *Phytophthora*, but localized in plastids of photosynthetic eukaryotes according to the original *Phytophthora* genome paper (Tyler et al., 2006) are indicated by asterisks in Target column. Bit scores of sequence matches from opisthokonts, amoebozoans, non-cyanobacterial bacteria or archaea are underlined if higher than those from photosynthetic stramenopiles, and in bold if higher than those from cyanobacteria and red algae*.

Of the 30 “algal” genes in *Phytophthora*, nine (30%) showed stronger BLAST matches (represented by higher bit scores) to homologs from opisthokonts, amoebozoans, non-cyanobacterial bacteria or archaea than to those from photosynthetic stramenopiles (**Table 1**). Another gene encoding methylthioadenosine phosphorylase (*P. ramorum* Gene ID 86425) had no detectable homologs in sequenced photosynthetic stramenopiles. Although the possibility of differential gene losses cannot be ruled out, genes with such a distribution pattern may also suggest an independent origin in oomycetes, such as horizontal gene transfer (HGT) from prokaryotes or other eukaryotes to oomycetes. Moreover, 16 of these 30 genes (about 53%) had more significant matches to homologs from opisthokonts, amoebozoans, non-cyanobacterial bacteria or archaea than to those from red algae and cyanobacteria (**Table 1**). Particularly, four genes had the strongest BLAST matches in non-cyanobacterial bacteria, and two in opisthokonts or amoebozoans. This observation based on simple pairwise comparisons suggests that many of these “algal” genes in oomycetes have no stronger similarity to photosynthetic stramenopile, red algal or cyanobacterial sequences. If sequence similarity is largely correlated with sequence relatedness, as commonly believed, the nature of these “algal” genes might be seriously questioned.

We further performed phylogenetic analyses on each of these 30 “algal” genes to evaluate its origin. If an oomycete gene is of red algal origin, the gene and its stramenopile (or chromalveolate) homologs are expected to form a clade sister to red algal and/or cyanobacterial sequences. This, however, was not the pattern uncovered in our study. Tree topologies for 21 (70%) genes were poorly supported overall (or the position of oomycete sequences couldn’t be confidently determined), thus providing no sufficient evidence for any evolutionary scenarios (Supplementary Materials). These poorly supported tree topologies might be caused by multiple issues, for example, insufficient phylogenetic signal or heterogeneity in evolutionary rates. Nevertheless, such topologies, combined with the information of phylogenetic distribution from BLAST search, should not be interpreted as evidence for a red algal origin of involved *Phytophthora* genes. The remaining genes had relatively well-resolved phylogenies and will be detailed in the following sections.

### Algal or Cyanobacterial Genes in Oomycetes

In our analyses, several of these “algal” genes indeed showed a close affinity with algal or cyanobacterial sequences. In addition, for 12 “algal” genes previously identified in *Phytophthora*, the protein products of their plant and/or algal homologs are localized in plastids (Tyler et al., 2006), as predicted by TargetP (Emanuelsson et al., 2000) (**Table 1**). It is well known that proteins of organelles-derived genes are often re-imported into the original organelles (mitochondria or plastids) to participate in related biochemical activities (Bogorad, 1975; Ellis, 1981; Weeden, 1981; Timmis et al., 2004). This information has been frequently used as supplemental evidence for genes of organellar origin. However, such affinity with algal/cyanobacterial sequences or functionality in other plastids might not necessarily support the suggestion of a historical red algal endosymbiont in the ancestral stramenopile.

**FIGURE 1:**
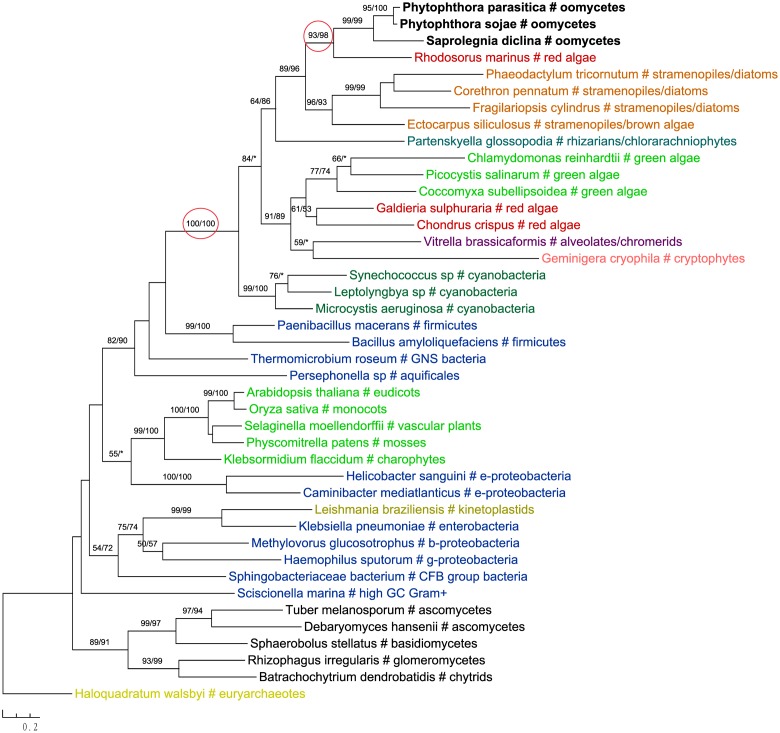
Molecular phylogeny of cobalamin-independent methionine synthase (MetE). Numbers above branches show bootstrap values in percentage for maximum likelihood and distance analyses, respectively. Values below 50% are indicated by asterisks.

**FIGURE 2:**
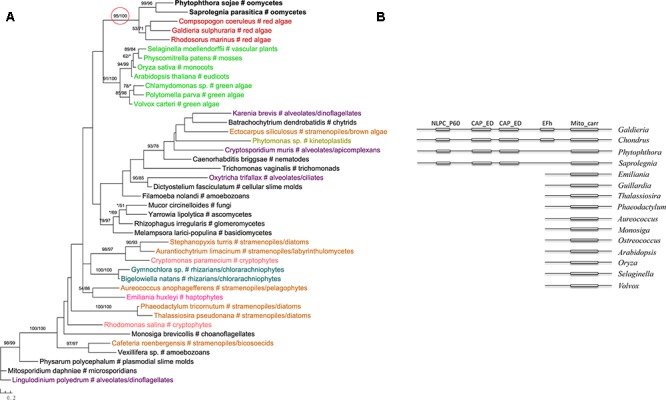
**(A)** Molecular phylogeny of cAMP-binding mitochondrial solute carrier. Numbers above branches show bootstrap values in percentage for maximum likelihood and distance analyses, respectively. Values below 50% are indicated by asterisks. **(B)** Schematic gene structure and domain composition of the cAMP-binding mitochondrial solute carrier gene in different lineages. Boxes show individual domains.

The most likely “algal” gene uncovered in our current study encodes cobalamin-independent methionine synthase (MetE). Our phylogenetic analyses indicated that *MetE* sequences from oomycetes, photosynthetic stramenopiles, chlorarachniophytes, chromerids and cryptophytes formed a large group with homologs of red algae, green algae, and cyanobacteria (**Figure 1**). Within this group, oomycete *MetE* sequences formed a strongly supported clade with red algal instead of other stramenopile homologs. Although the overall molecular phylogeny of *MetE* is consistent with an algal origin of oomycetes and, to a certain extent, an algal/cyanobacterial origin of all stramenopiles, the strength of this evidence is somewhat compromised by the fact that oomycete and other stramenopile sequences didn’t form a monophyletic group (see Discussion). Two other similar cases are related to the genes encoding prolyl oligopeptidase II (Supplementary Figure 1) and cAMP-binding mitochondrial solute carrier (**Figure 2**). For both genes, their molecular phylogenies showed that oomycete and red algal sequences were closely related. Particularly in the latter case, a NLPC_P60 and two CAP_ED domains are uniquely shared by oomycetes and red algae, but are absent from other stramenopiles (**Figure 2**). A parsimonious explanation for these findings would be that oomycetes obtained this gene from red algae directly or vice versa.

**FIGURE 3:**
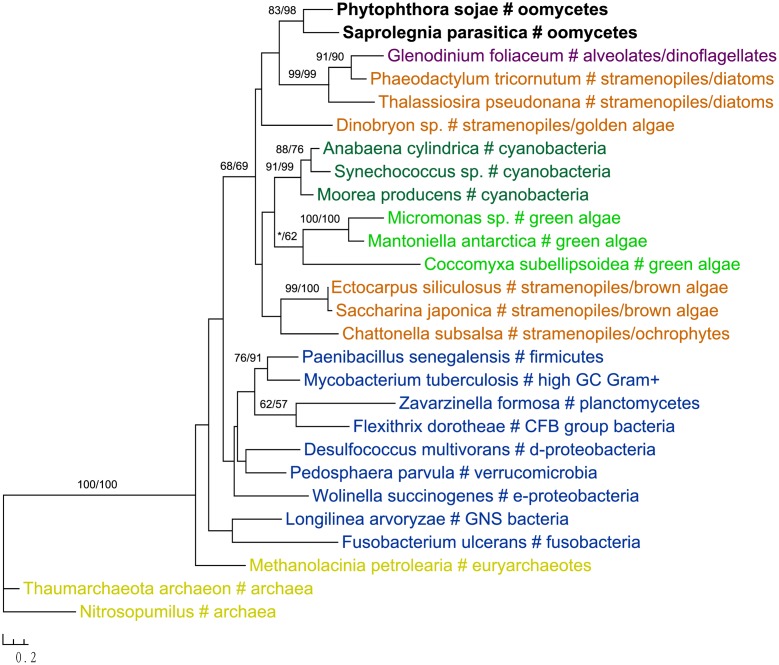
Molecular phylogeny of NCAIR mutase. Numbers above branches show bootstrap values in percentage for maximum likelihood and distance analyses, respectively. Values below 50% are indicated by asterisks.

Two genes in our analyses were found to be specifically related to green plant sequences, which is in disagreement with the suggestion of a red algal plastid in the ancestral stramenopile. The gene encoding NCAIR mutase does not have detectable homologs in red algae. Phylogenetic analyses of NCAIR mutase supported a monophyletic group including sequences from oomycetes, photosynthetic stramenopiles, dinoflagellates, green algae and cyanobacteria (**Figure 3**). Because of the lack of detectable NCAIR mutase homologs in red algae, a red algal origin of this gene in all stramenopiles cannot be concluded. On the other hand, an independent green algal endosymbiont in stramenopiles might potentially explain such a distribution pattern (Moustafa et al., 2009; Dorrell and Smith, 2011). The other green plants-related gene in oomycetes encodes a probable folate-biopterin transporter (Supplementary Figure 22). Our analyses showed that sequences from oomycetes, diatom *Thalassionema frauenfeldii* and land plants formed a strongly supported clade, whereas other photosynthetic sequences, including red algae and cyanobacteria, formed another large group with only modest support.

**FIGURE 4:**
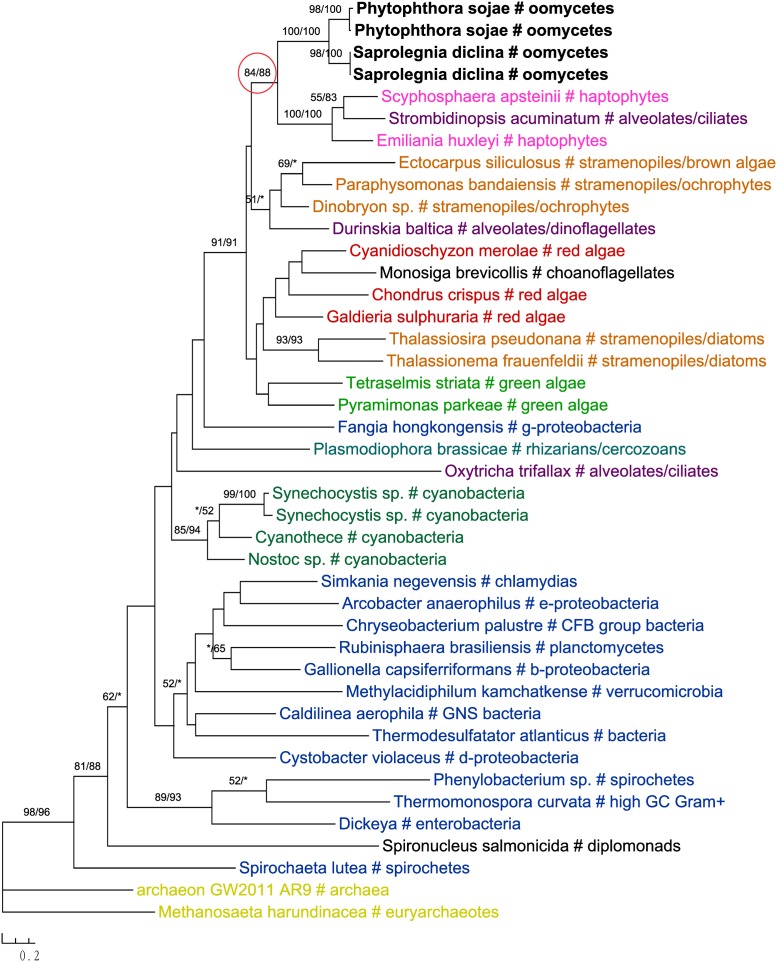
Molecular phylogeny of glucokinase. Numbers above branches show bootstrap values in percentage for maximum likelihood and distance analyses, respectively. Values below 50% are indicated by asterisks.

**FIGURE 5:**
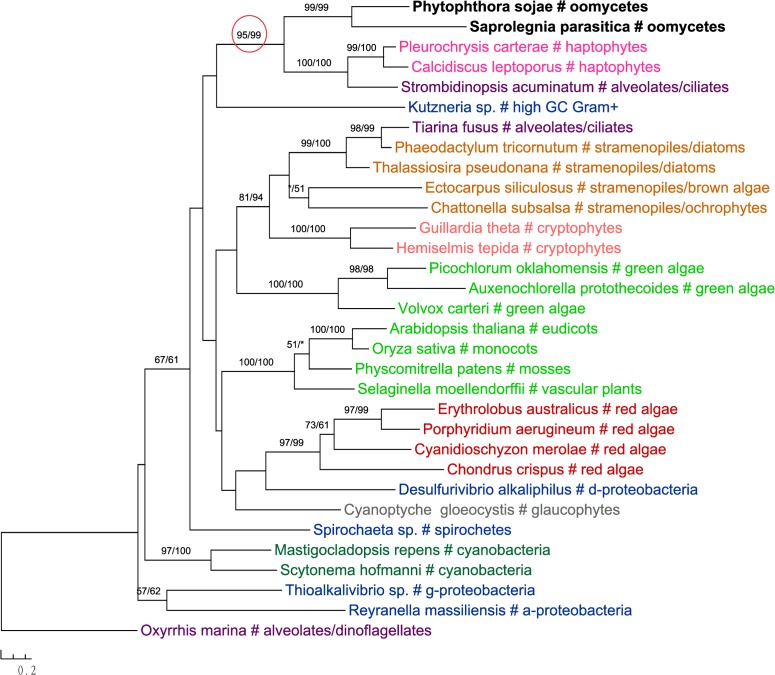
Molecular phylogeny of ketol-acid reductoisomerase. Numbers above branches show bootstrap values in percentage for maximum likelihood and distance analyses, respectively. Values below 50% are indicated by asterisks.

In addition to primary algae and cyanobacteria, several groups of eukaryotes that have secondary plastids through higher-order endosymbioses might also be potential donors for genes in oomycetes. For instance, phylogenetic analyses of glucokinase indicated that sequences from oomycetes, haptophytes and ciliates formed a well-supported clade, which in turn grouped with homologs from photosynthetic stramenopiles, red algae, green algae, choanoflagellates and dinoflagellates (**Figure 4**). A similar case was also observed for the gene encoding ketol-acid reductoisomerase (**Figure 5**). As it is known that ciliates contain sequences of algal origin (Reyes-Prieto et al., 2008), this topology might suggest HGT from haptophytes-related groups to oomycetes, and again provides no support for a common photosynthetic origin between oomycetes and other stramenopiles.

### Other Potential Evolutionary Scenarios

As indicated above, a large fraction of “algal” genes in oomycetes/stramenopiles showed stronger matches in our BLAST search to homologs from opisthokonts, amoebozoans or non-cyanobacterial bacteria rather than those from red algae and cyanobacteria. For several of these genes, this relationship was also confirmed by subsequent phylogenetic analyses.

**FIGURE 6:**
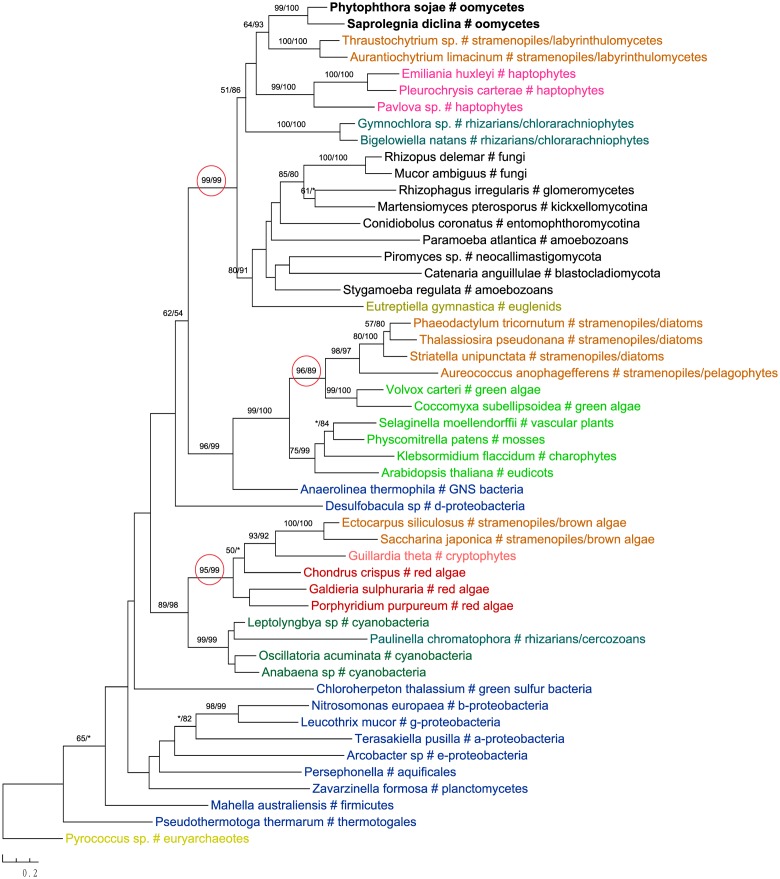
Molecular phylogeny of 2-isopropylmalate synthase. Numbers above branches show bootstrap values in percentage for maximum likelihood and distance analyses, respectively. Values below 50% are indicated by asterisks.

One of these genes encodes 2-isopropylmalate synthase in leucine biosynthesis and was previously detailed in the *Phytophthora* genome paper (Tyler et al., 2006). According to the authors, this gene was subject to at least two transfer events in eukaryotes: sequences of primary photosynthetic eukaryotes and stramenopiles (including oomycetes) were derived from cyanobacteria, whereas sequences of fungi were from α-proteobacteria. Specifically, diatom sequences were found to group with green plant rather than red algal homologs, which was interpreted as a separate ancestry or artifacts due to incomplete sampling (Tyler et al., 2006). Our current analyses support the previous conclusion that this gene in stramenopiles might have different origins, but also suggest a potentially more complicated evolutionary scenario. While sequences from brown algae and cryptophytes indeed grouped with red algal homologs, those from diatoms and *Aureococcus* with green plant sequences instead (**Figure 6**). The relationships between brown algae, cryptophytes and red algae uncovered here is in line with the suggestion of serial endosymbioses by Stiller et al. (2014), where a red alga was first adopted by a cryptophyte that was in turn engulfed by ochrophytes. The sequence affiliation between diatoms, *Aureococcus* and green algae might point to separate origins of this gene in other photosynthetic stramenopiles [e.g., from a potential green algal endosymbiont (Moustafa et al., 2009; Dorrell and Smith, 2011) or an independent HGT event]. Nevertheless, unlike previously reported in the *Phytophthora* genome paper (Tyler et al., 2006), oomycete sequences grouped with labyrinthulomycetes, another group of heterotrophic stramenopiles, and other eukaryotes, rather than being affiliated with diatoms, primary photosynthetic eukaryotes and cyanobacteria (**Figure 6**).

**FIGURE 7:**
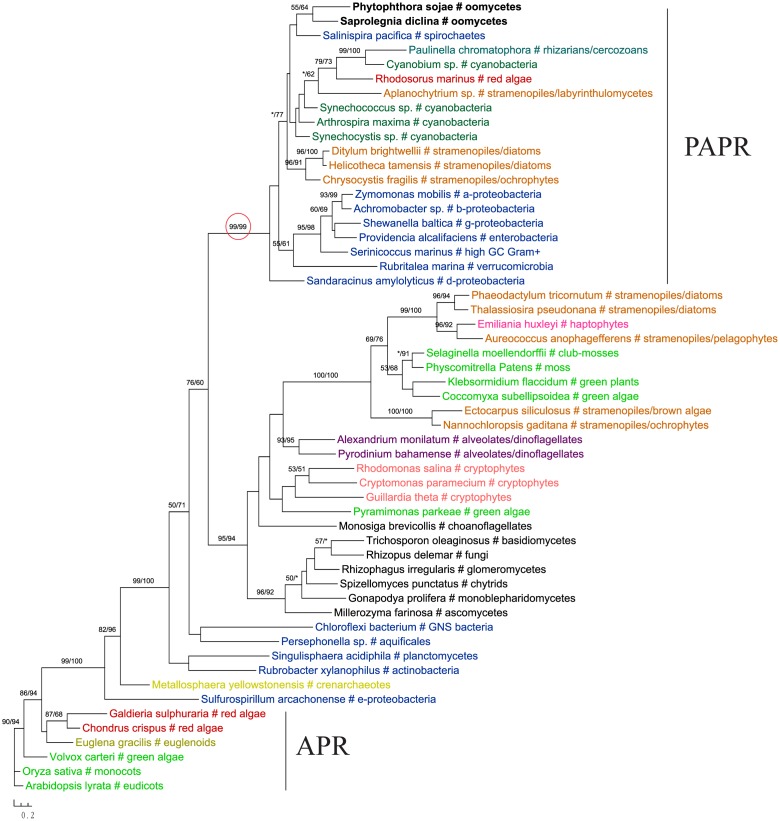
Molecular phylogeny of 3′-phosphoadenosine 5′-phosphosulfate reductase (PAPR) and adenosine 5′-phosphosulfate reductase (APR). Numbers above branches show bootstrap values in percentage for maximum likelihood and distance analyses, respectively. Values below 50% are indicated by asterisks.

The gene encoding 3′-phosphoadenosine 5′-phosphosulfate reductase (PAPR), an enzyme in the sulfate assimilation pathway, is another example highlighting the potential pitfalls of insufficient sampling. PAPR and adenosine 5′-phosphosulfate reductase (APR) are homologous proteins and have a complex evolutionary history in eukaryotes (Kopriva et al., 2002; Kopriva and Koprivova, 2004; Patron et al., 2008). The *APR* gene was previously thought to exist in land plants, algae, and phototrophic bacteria. *PAPR*, on the other hand, was initially identified mainly in fungi and bacteria (Kopriva et al., 2002). Several more recent studies reported *PAPR* sparely in phototrophic eukaryotes, suggesting potential HGT events (Kopriva and Koprivova, 2004; Kopriva et al., 2007; Patron et al., 2008). Particularly, the study of Patron et al. (2008) indicated a potential bacterial origin of *PAPR* in *P. sojae*. With a much larger taxonomic sampling, our analyses showed that sequences from some stramenopiles (including oomycetes), bacteria (both cyanobacteria and non-cyanobacteria) and *Paulinella chromatophora* formed a major *PAPR* clade (**Figure 7**). The cyanobacterial origin of *PAPR* in *P. chromatophora* is somewhat expected, as this species contains an independently evolved plastid organelle (cyanobacterial endosymbiont) (Marin et al., 2005). As the overall topology of this clade is poorly supported, whether *PAPR* in stramenopiles was derived from a red algal endosymbiont or a separate HGT event could not be answered by our study.

## Discussion

As evidence of historical plastids in oomycetes, the “algal” genes identified in *Phytophthora* genomes were used to support a common photosynthetic ancestry of stramenopiles, and the chromalveolate hypothesis in general. In the *Phytophthora* genome paper (Tyler et al., 2006), the identification of “algal” genes was heavily based on significant matches to sequences from red algae or cyanobacteria. Because the identification of foreign genes in eukaryote can be affected by taxonomic samplings and methods of analyses, studies on algal genes in different eukaryotes sometimes led to different interpretations after re-analyses. For example, 263 red algal genes and 250 green plant genes were reported in *Chromera velia* (Woehle et al., 2011), but only 23 and nine of them, respectively, were confirmed after re-evaluation (Burki et al., 2012a). When more stringent criteria were applied, the number of putative green algal genes in diatoms decreased from 1,700 (Moustafa et al., 2009) to only 144 (Dorrell and Smith, 2011). While an algal endosymbiont in the common ancestor of stramenopiles or any other lineages could certainly be a significant source of foreign genes, other issues, notably phylogenetic artifacts, insufficient sampling, differential gene losses and independent HGT events, could all lead to the same or similar atypical gene distributions or relationships.

With a much larger sampling and careful phylogenetic analyses, we revisited the 30 most likely “algal” genes identified in the *Phytophthora* genomes (Tyler et al., 2006). Our results show that the identification of these “algal” genes, to a great extent, was affected by limited genome data then available for certain eukaryotic lineages. Almost none of these 30 genes confidently supports the hypothesis of a red algal endosymbiont in the common ancestor of stramenopiles. Although the molecular phylogeny of *MetE* is indeed consistent with the suggestion of a photosynthetic ancestry of stramenopiles, its topology does not strictly support a historical red plastid in this lineage. As such, our current study is largely consistent with the statistical genome analyses of Stiller et al. (2009), which found no evidence for a red algal endosymbiont in the ancestral stramenopile. However, we should also caution here that, because the parasitic nature of oomycetes, the possibility of plastid loss during oomycete evolution cannot be entirely excluded based on our data. Furthermore, given the fact that many of the sampled sequences in our analyses were from transcriptomic data, it is unclear whether and how the data quality, for example potential sequencing contamination, might have affected our results. Additional investigations are needed to resolve this significant, nevertheless controversial, issue of eukaryotic evolution.

On the other hand, our results also indicate that the abnormal phylogenetic signal of these “algal” genes might be caused by a complex evolutionary history of oomycetes or stramenopiles. Although the origins of these 30 genes in oomycetes or stramenopiles are not always clear, several of them were found to be related to miscellaneous algae. To a certain extent, such sequence relatedness to various lineages might be attributed to other potential historical endosymbioses or independent HGT events involving oomycetes or stramenopiles. For instance, in lieu of the chromalveolate hypothesis, serial endosymbioses between different photosynthetic lineages have been proposed to explain the evolution of red algal plastids (Sanchez-Puerta and Delwiche, 2008; Stiller et al., 2014). A potential green algal endosymbiont was also suggested in stramenopiles (Moustafa et al., 2009; Dorrell and Smith, 2011). Furthermore, horizontally acquired genes have been reported in different eukaryotic lineages (Richardson and Palmer, 2007; Keeling and Palmer, 2008; Andersson, 2009; Dunning Hotopp, 2011; Huang and Yue, 2012; Soucy et al., 2015; Qiu et al., 2016), even though some of the earlier reports might turn out to be false positives as suggested by our current study. Especially for microbial eukaryotes, the importance and frequency of HGT in their evolution is increasingly being appreciated (Keeling and Palmer, 2008; Andersson, 2009), and there is evidence that microbial eukaryotes might have frequently acquired genes from various organisms, instead of a specific source of endosymbiotic relationship (Huang et al., 2004; Loftus et al., 2005; Carlton et al., 2007; Bowler et al., 2008; Sun et al., 2010; Yue et al., 2013). Oomycetes originated in marine environments and gradually spread to freshwater and terrestrial environments (Beakes and Sekimoto, 2009; Beakes et al., 2012). Bacteria, miscellaneous algae or other organisms in a common habitat could be potential sources of foreign genes in oomycetes. Additionally, feeding activities of their ancestors in aquatic environments or the parasitic feature of modern species [many oomycetes are parasites; for instance, the early diverging species *Eurychasma dicksonii* is an obligate parasite of marine brown algae (Küpper and Müller, 1999; Gachon et al., 2009; Strittmatter et al., 2009)] might have also facilitated genes acquisition in oomycetes. Indeed, several studies have already reported gene acquisition events in oomycetes and other stramenopiles, including fungi to oomycetes (Richards et al., 2006, 2011), bacteria to diatoms (Bowler et al., 2008), and different prokaryotic or eukaryotic sources to *Blastocystis* (Tsaousis et al., 2012; Eme et al., 2017). In this regard, our finding of multiple foreign genes in oomycetes might reflect the interactions among red/green algae, oomycetes/stramenopiles, and other microbes, as well as their ensuing genetic integration.

## Author Contributions

JH conceived the study and wrote the manuscript. QW performed the analyses and wrote the manuscript. HS contributed to the analyses.

## Conflict of Interest Statement

The authors declare that the research was conducted in the absence of any commercial or financial relationships that could be construed as a potential conflict of interest.

## Footnotes

1 http://genome.jgi.doe.gov/programs/fungi/index.jsf
