# Supplementary material for: Re-analyses of “Algal” Genes Suggest a Complex Evolutionary History of Oomycetes
Source: Front Plant Sci. 2017 Sep 6;8:1540. doi: 10.3389/fpls.2017.01540 (PMC5592239; doi:10.3389/fpls.2017.01540)
Supplement: Supplementary file 1 [file Supplementary_Figures_Tables.DOCX]

Supplementary Material

Re-analyses of “algal” genes suggest a complex evolutionary history of oomycetes

Qia Wang, Hang Sun*, Jinling Huang*

*** Correspondence:**

Hang Sun
sunhang@mail.kib.ac.cn

Jinling Huang
[huangj@ecu.edu](mailto:huangj@ecu.edu)

## Supplementary Figures


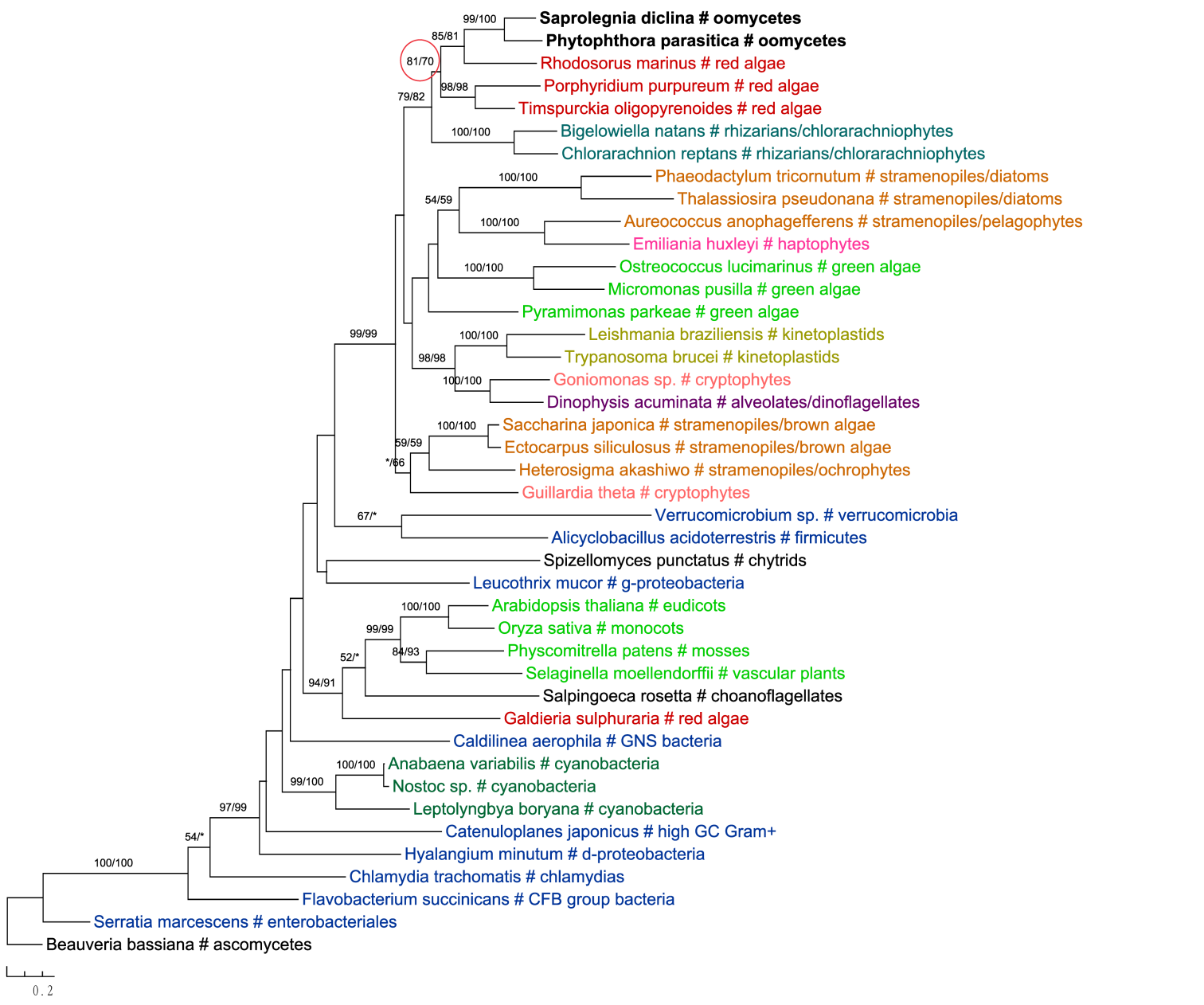


**Supplementary Figure 1.** Molecular phylogeny of prolyl oligopeptidase II. Numbers above branches show bootstrap values in percentage for maximum likelihood and distance analyses, respectively. Values below 50% are indicated by asterisks.


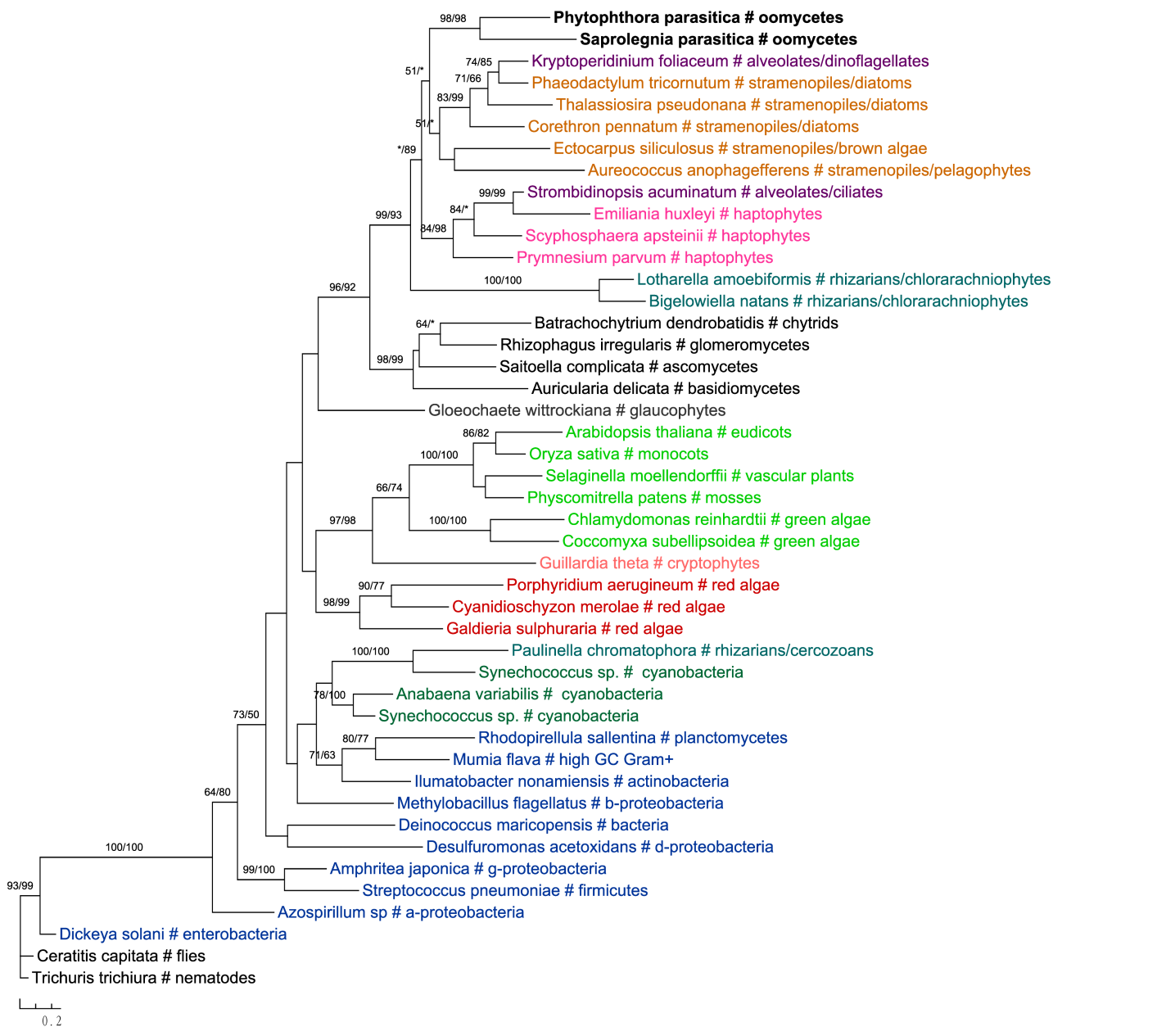


**Supplementary Figure 2.** Molecular phylogeny of threonine ammonia-lyase. Numbers above branches show bootstrap values in percentage for maximum likelihood and distance analyses, respectively. Values below 50% are indicated by asterisks.


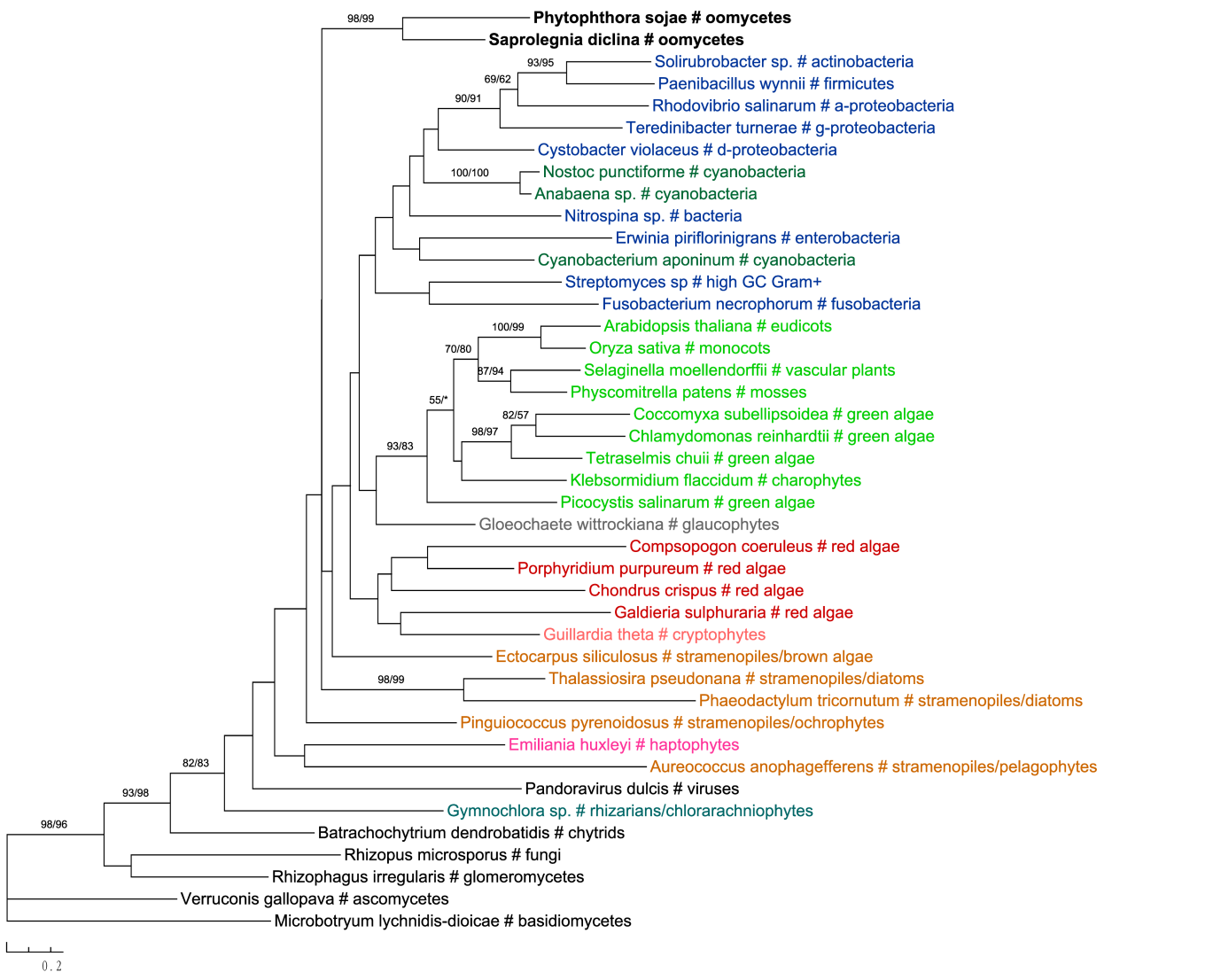


**Supplementary Figure 3.** Molecular phylogeny of anthranilate synthase. Numbers above branches show bootstrap values in percentage for maximum likelihood and distance analyses, respectively. Values below 50% are indicated by asterisks.


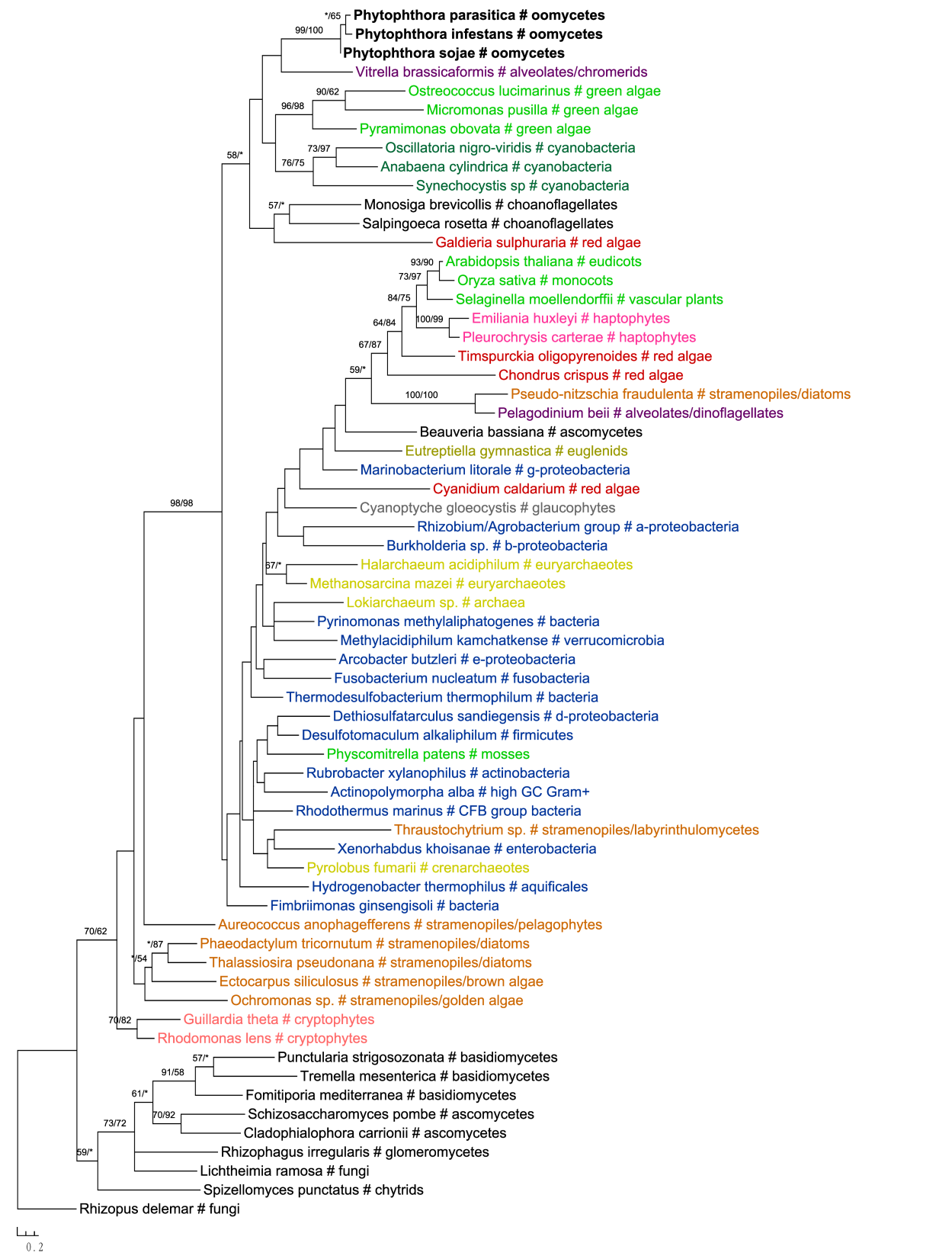


**Supplementary Figure 4.** Molecular phylogeny of uroporphyrinogen-III methyltransferase. Numbers above branches show bootstrap values in percentage for maximum likelihood and distance analyses, respectively. Values below 50% are indicated by asterisks.


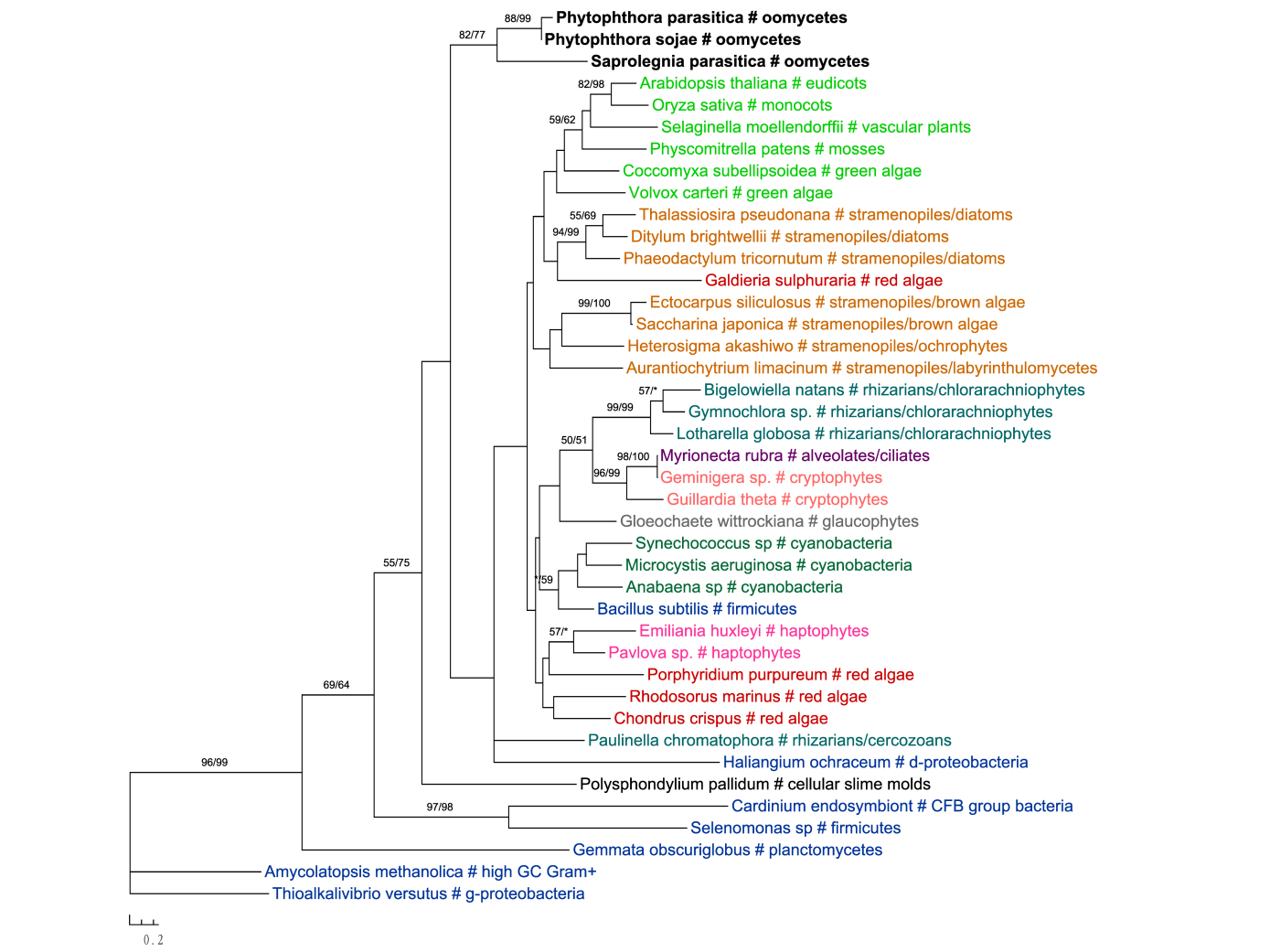


**Supplementary Figure 5.** Molecular phylogeny of tRNA (guanine-N(7)-)-methyltransferase-like. Numbers above branches show bootstrap values in percentage for maximum likelihood and distance analyses, respectively. Values below 50% are indicated by asterisks.


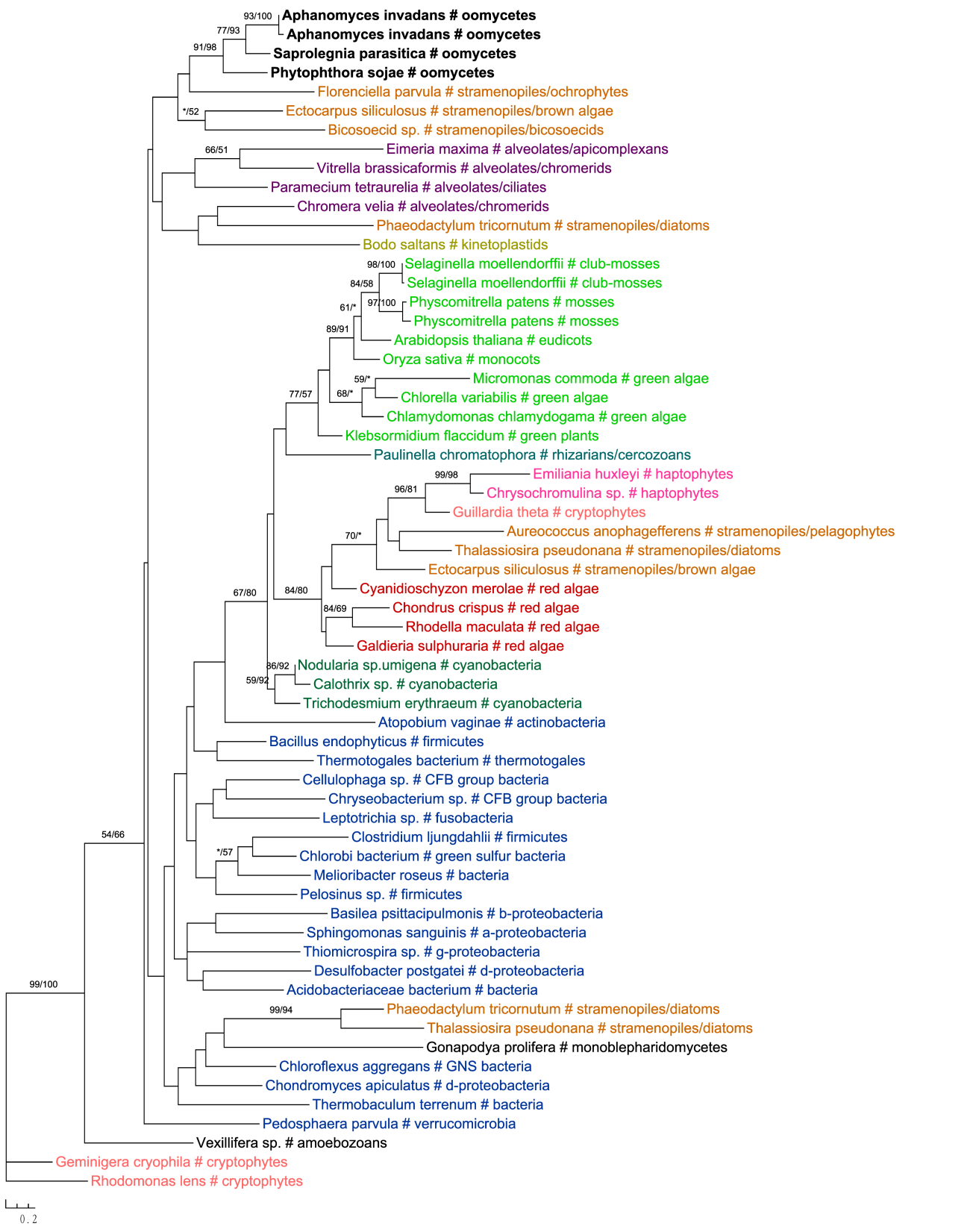


**Supplementary Figure 6.** Molecular phylogeny of phosphatidate cytidylyltransferase. Numbers above branches show bootstrap values in percentage for maximum likelihood and distance analyses, respectively. Values below 50% are indicated by asterisks.

**
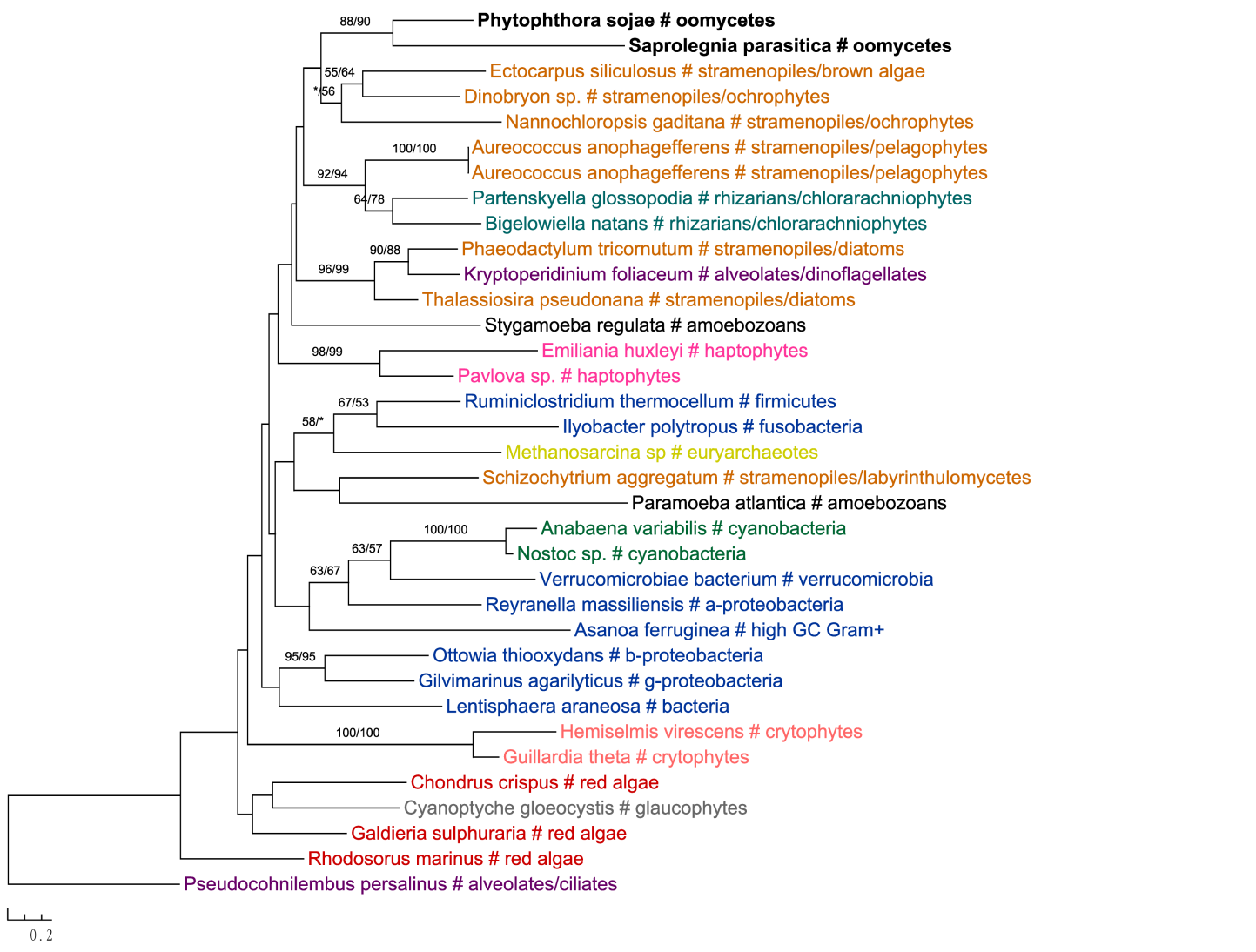
**

**Supplementary Figure 7.** Molecular phylogeny of phosphoserine aminotransferase. Numbers above branches show bootstrap values in percentage for maximum likelihood and distance analyses, respectively. Values below 50% are indicated by asterisks.


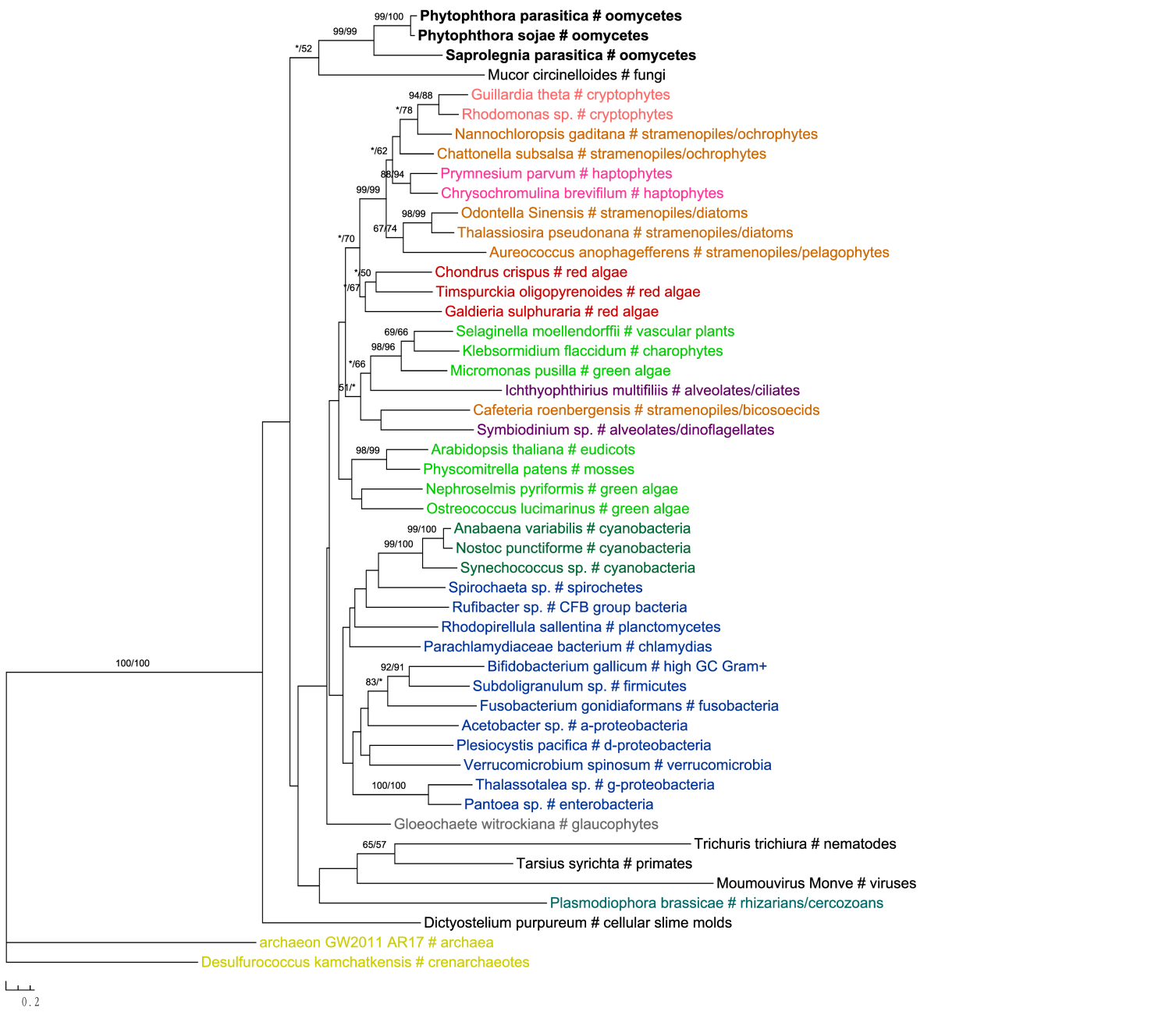


**Supplementary Figure 8.** Molecular phylogeny of asparaginyl tRNA synthetase. Numbers above branches show bootstrap values in percentage for maximum likelihood and distance analyses, respectively. Values below 50% are indicated by asterisks.


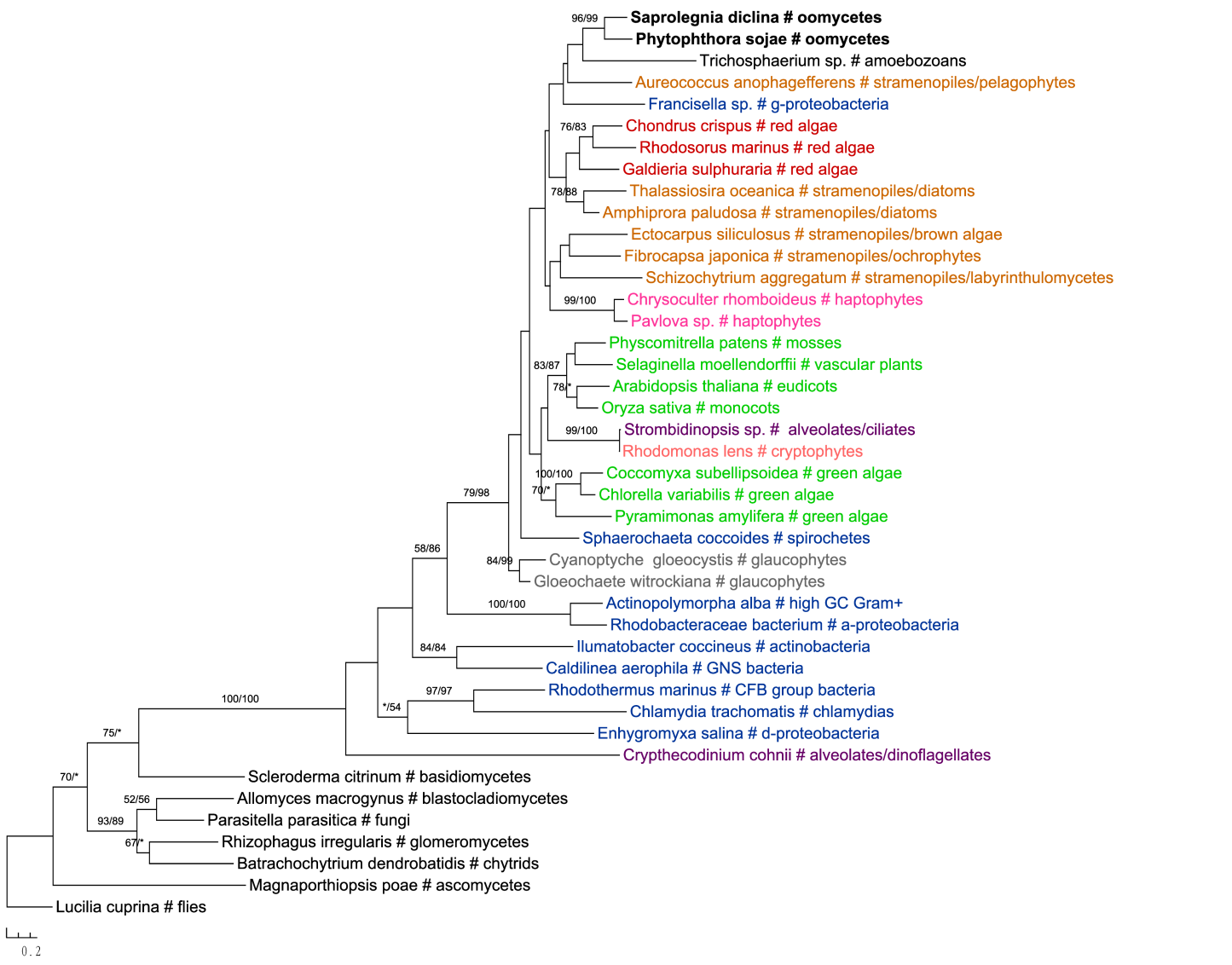


**Supplementary Figure 9.** Molecular phylogeny of SAICAR synthetase. Numbers above branches show bootstrap values in percentage for maximum likelihood and distance analyses, respectively. Values below 50% are indicated by asterisks.


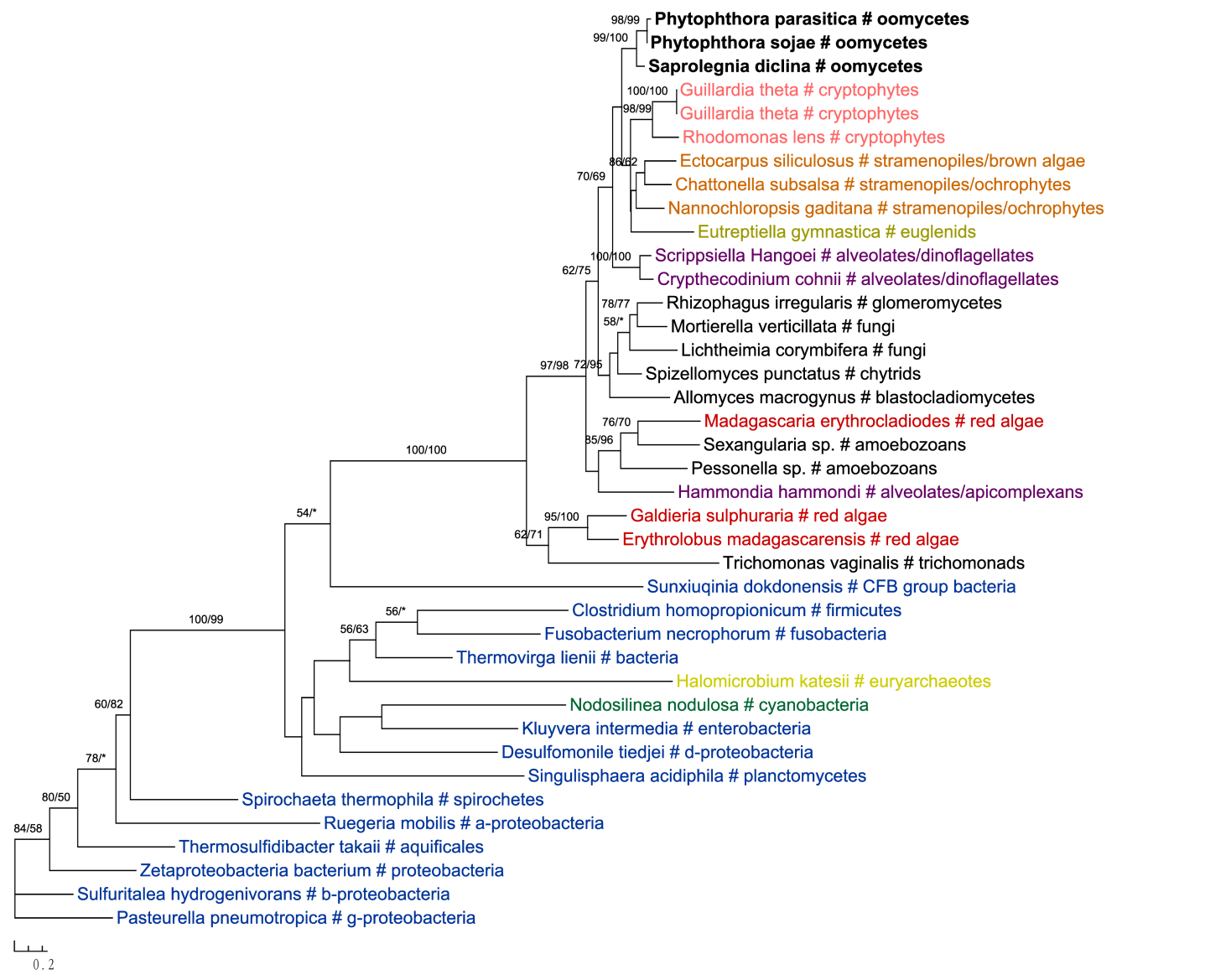


**Supplementary Figure 10.** Molecular phylogeny of histidinol-phosphate aminotransferase. Numbers above branches show bootstrap values in percentage for maximum likelihood and distance analyses, respectively. Values below 50% are indicated by asterisks.


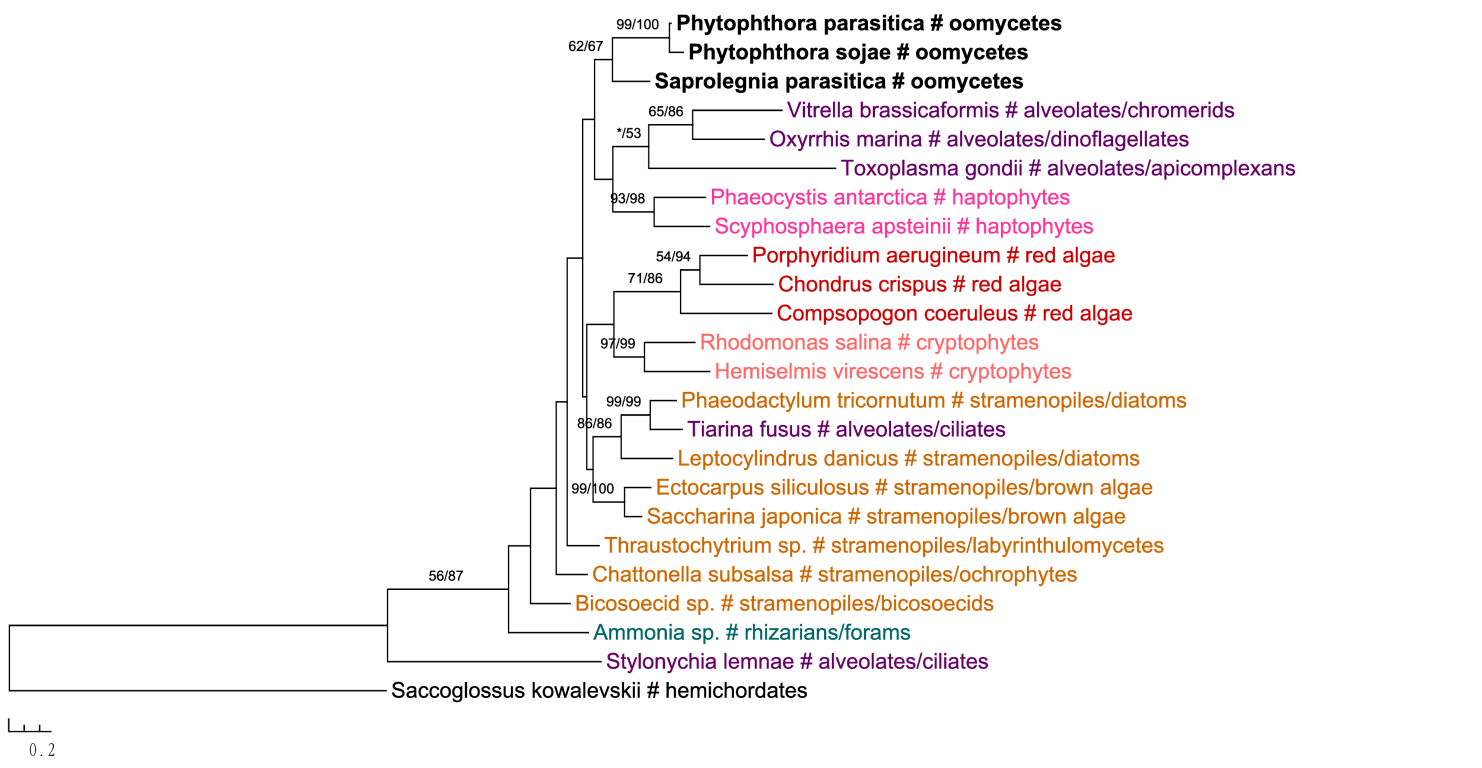


**Supplementary Figure 11.** Molecular phylogeny of zinc carboxypeptidase A. Numbers above branches show bootstrap values in percentage for maximum likelihood and distance analyses, respectively. Values below 50% are indicated by asterisks.


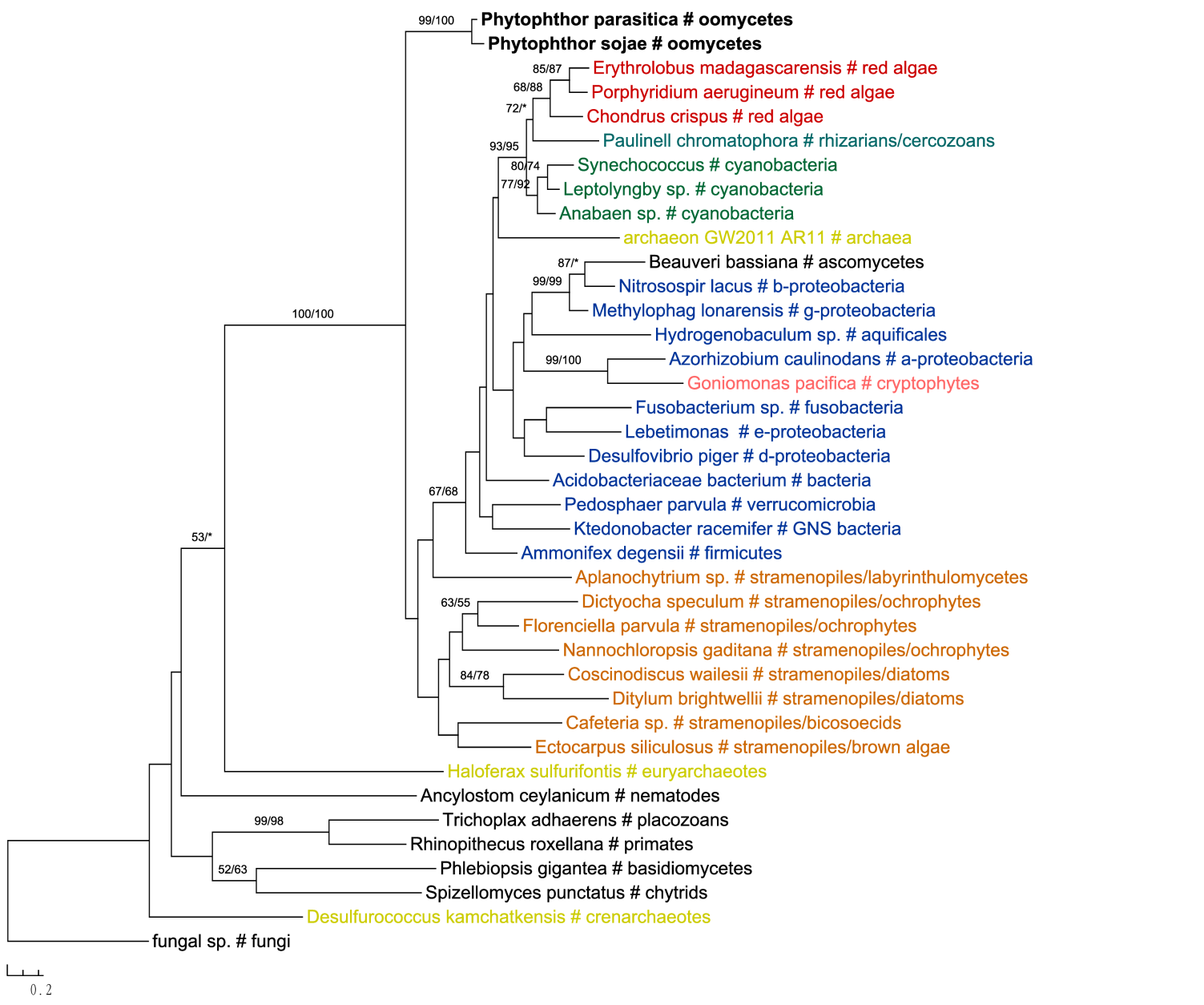


**Supplementary Figure 12.** Molecular phylogeny of enoyl-(acyl-carrier-protein) reductase. Numbers above branches show bootstrap values in percentage for maximum likelihood and distance analyses, respectively. Values below 50% are indicated by asterisks.


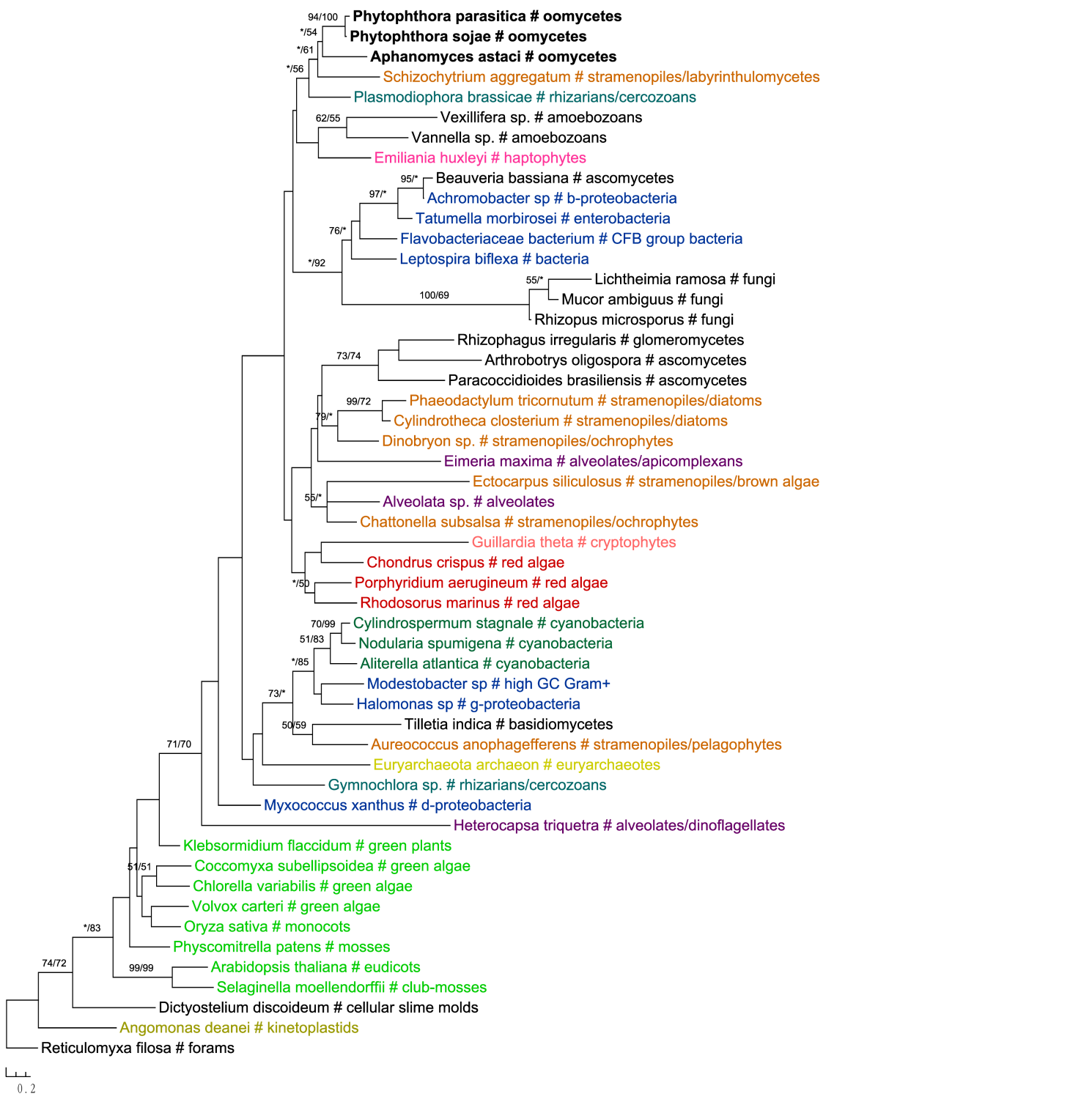


**Supplementary Figure 13.** Molecular phylogeny of sulfur transferase + methyl transferase fusion. Numbers above branches show bootstrap values in percentage for maximum likelihood and distance analyses, respectively. Values below 50% are indicated by asterisks.

**
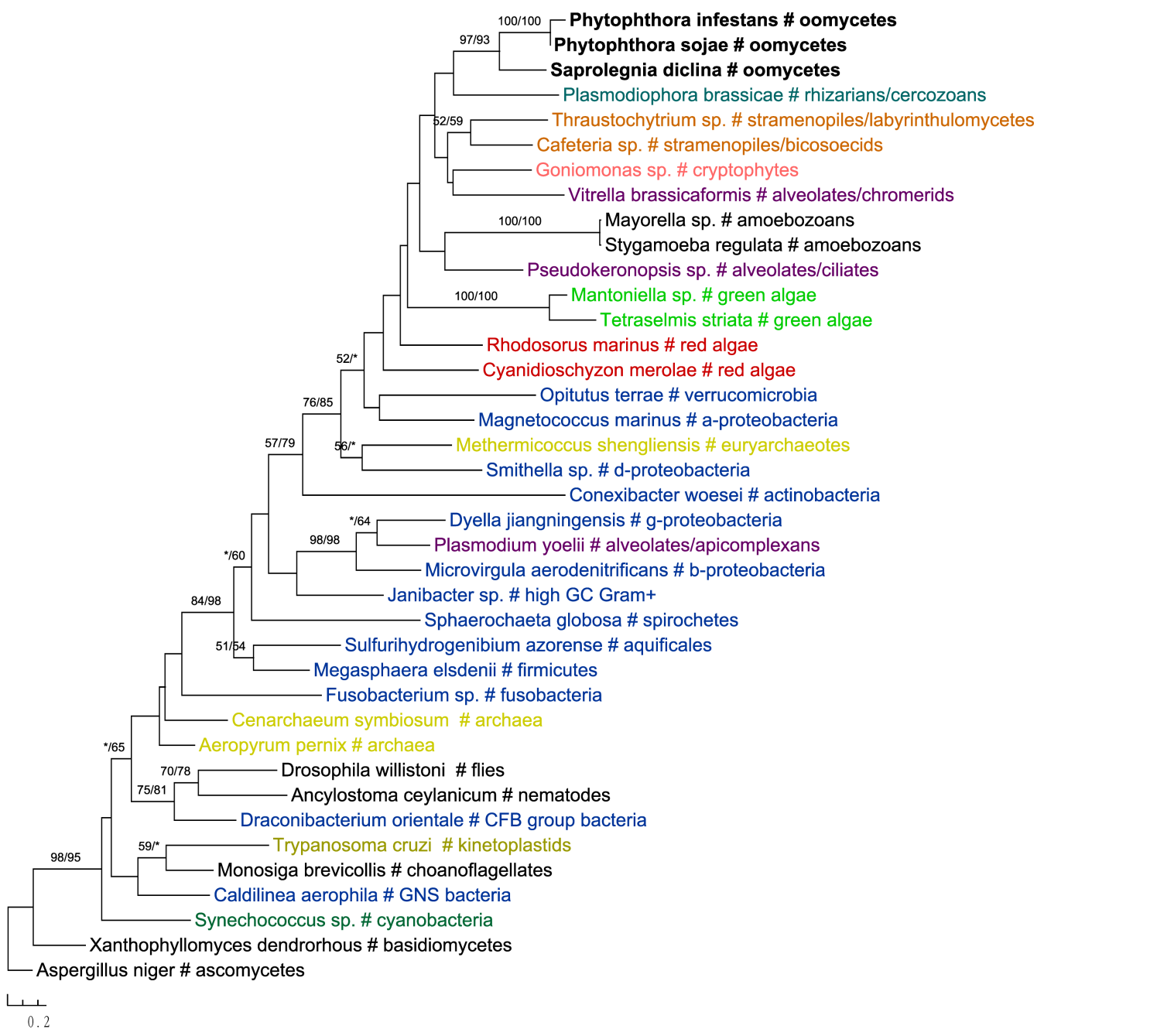
**

**Supplementary Figure 14.** Molecular phylogeny of methylthioadenosine phosphorylase. Numbers above branches show bootstrap values in percentage for maximum likelihood and distance analyses, respectively. Values below 50% are indicated by asterisks.


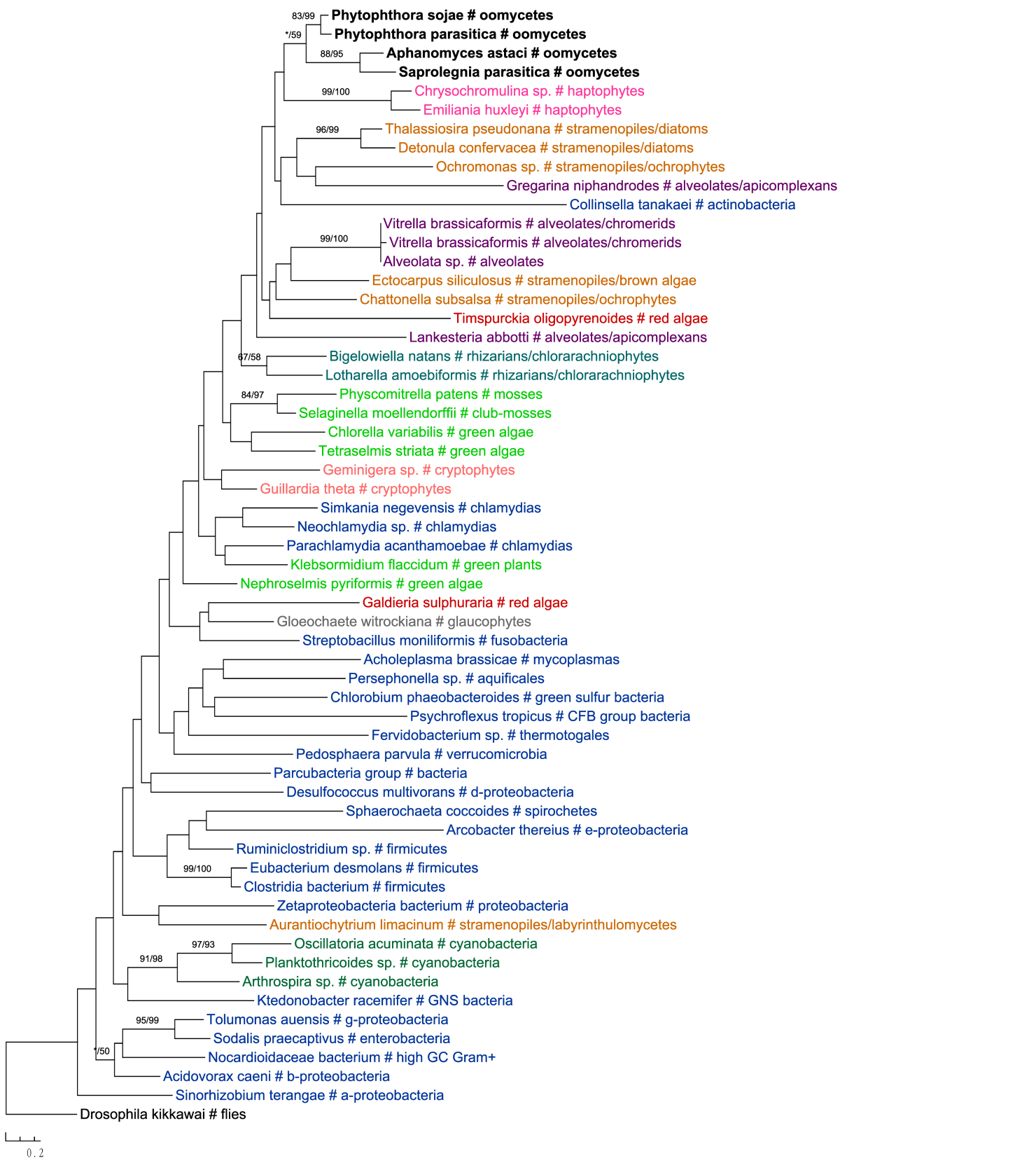


**Supplementary Figure 15.** Molecular phylogeny of ribonuclease HII. Numbers above branches show bootstrap values in percentage for maximum likelihood and distance analyses, respectively. Values below 50% are indicated by asterisks.


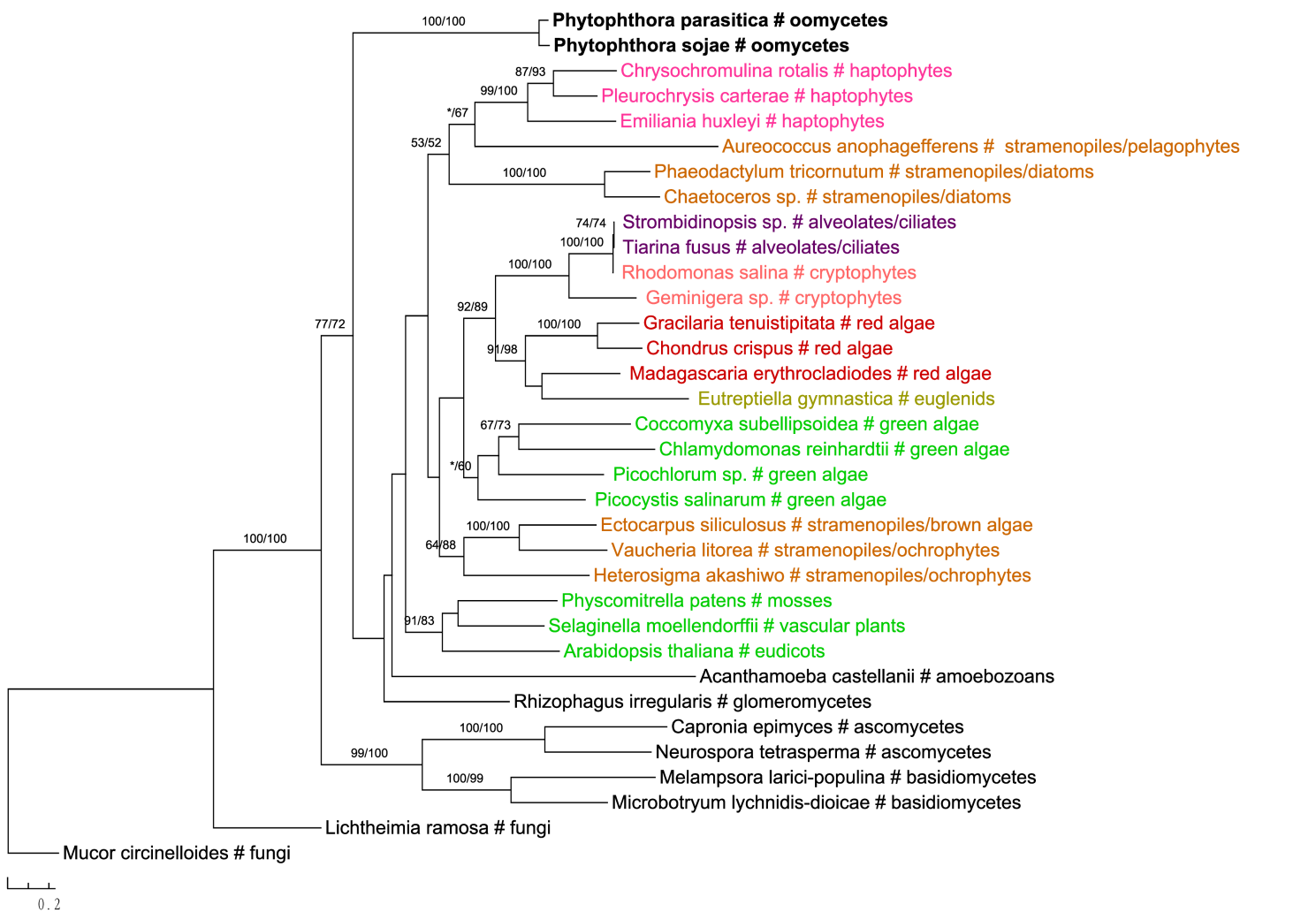


**Supplementary Figure 16.** Molecular phylogeny of nitrate reductase. Numbers above branches show bootstrap values in percentage for maximum likelihood and distance analyses, respectively. Values below 50% are indicated by asterisks.


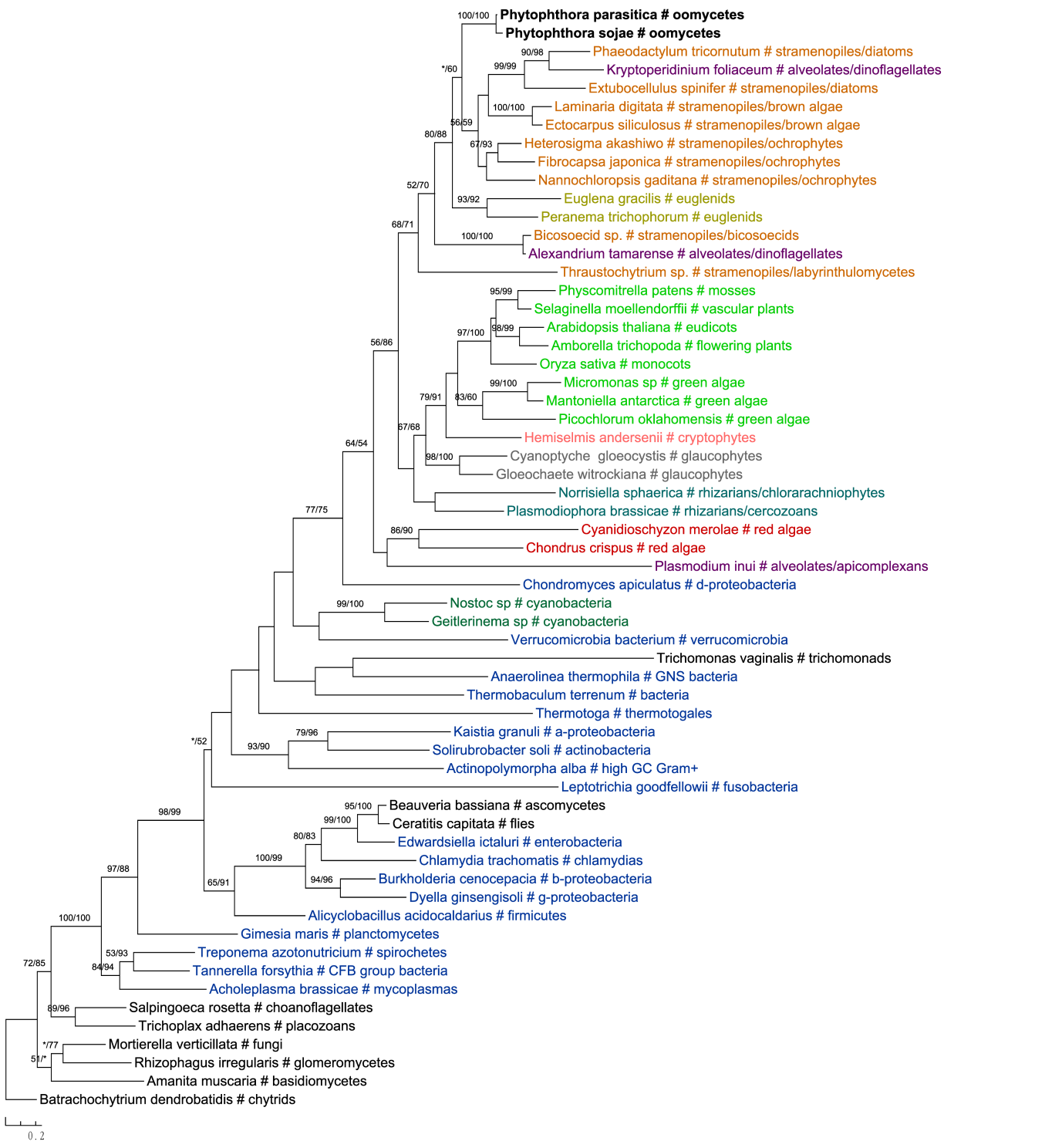


**Supplementary Figure 17.** Molecular phylogeny of 6-phosphogluconate dehydrogenase. Numbers above branches show bootstrap values in percentage for maximum likelihood and distance analyses, respectively. Values below 50% are indicated by asterisks.


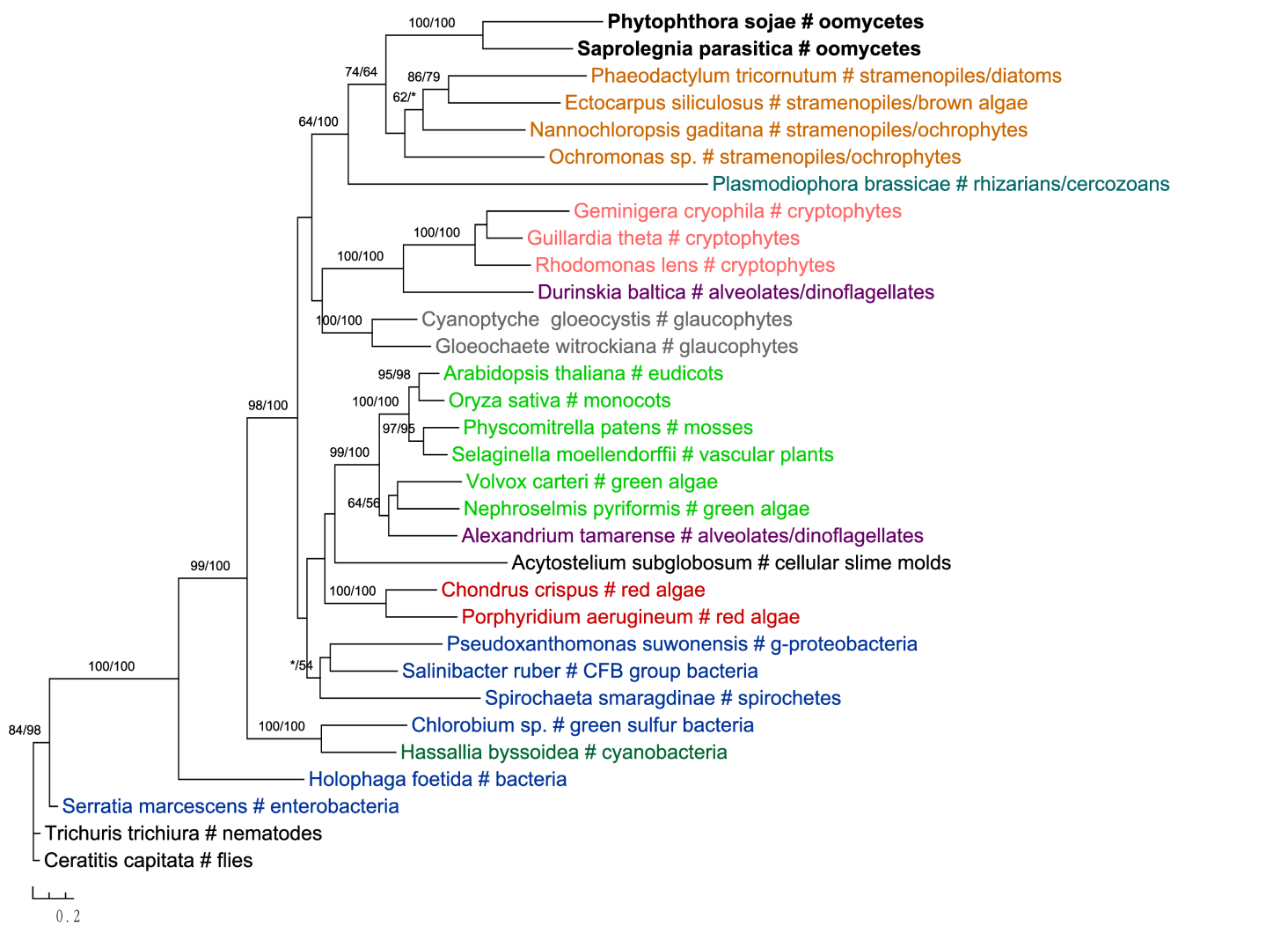


**Supplementary Figure 18.** Molecular phylogeny of aspartate kinase/homoserine dehydrogenase. Numbers above branches show bootstrap values in percentage for maximum likelihood and distance analyses, respectively. Values below 50% are indicated by asterisks.


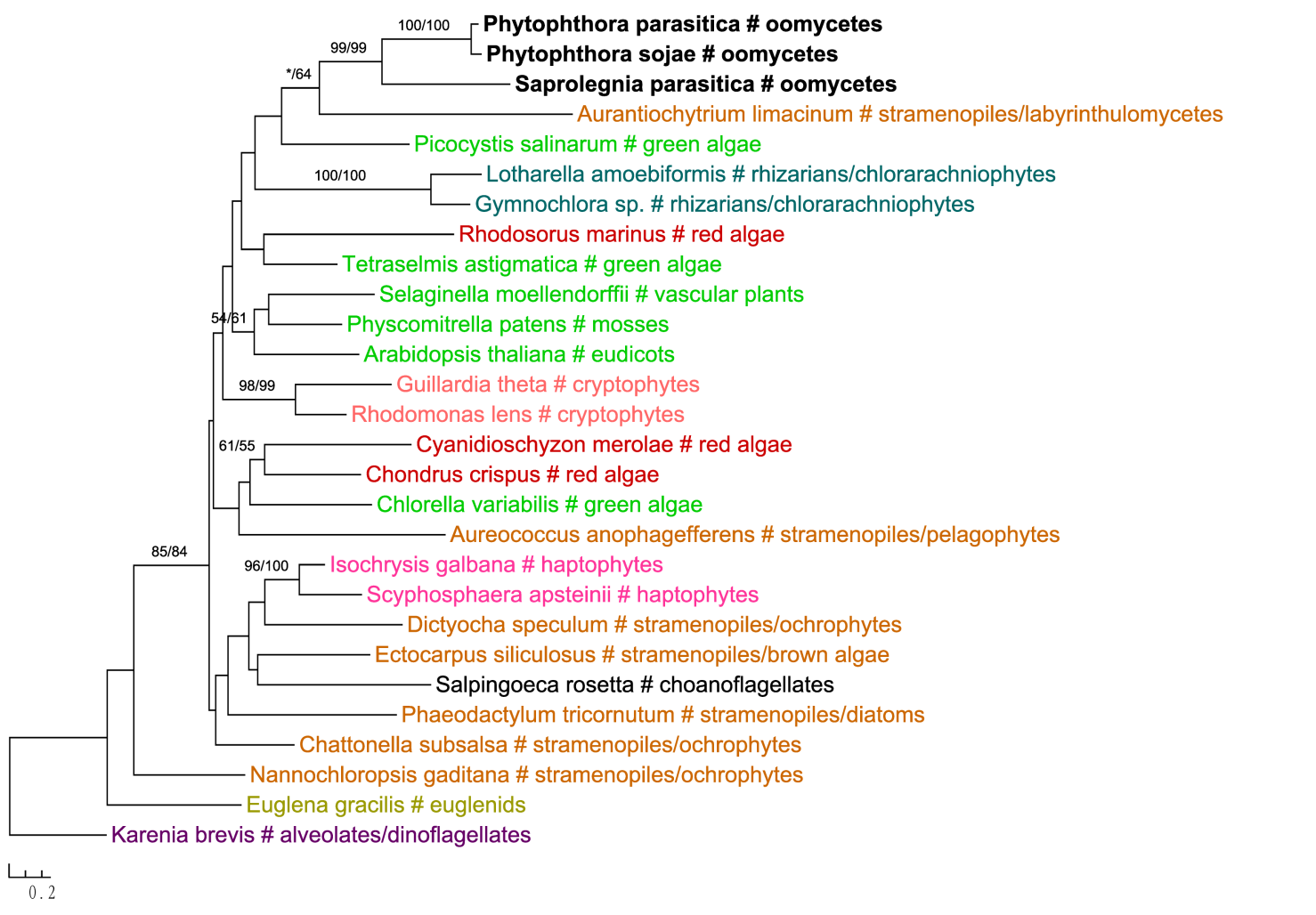


**Supplementary Figure 19.** Molecular phylogeny of galactonolactone dehydrogenase. Numbers above branches show bootstrap values in percentage for maximum likelihood and distance analyses, respectively. Values below 50% are indicated by asterisks.


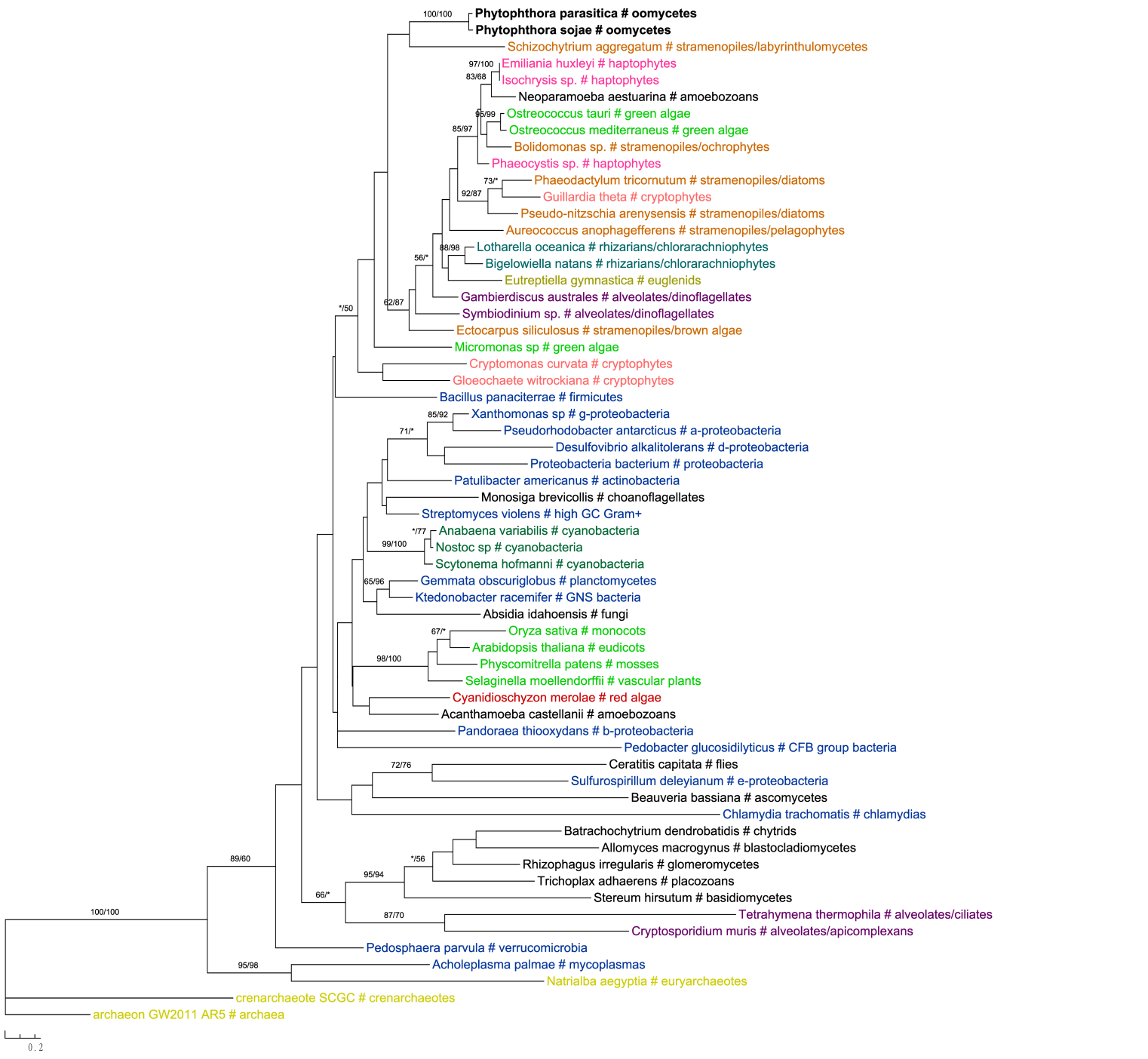


**Supplementary Figure 20.** Molecular phylogeny of cobalamin synthesis protein. Numbers above branches show bootstrap values in percentage for maximum likelihood and distance analyses, respectively. Values below 50% are indicated by asterisks.


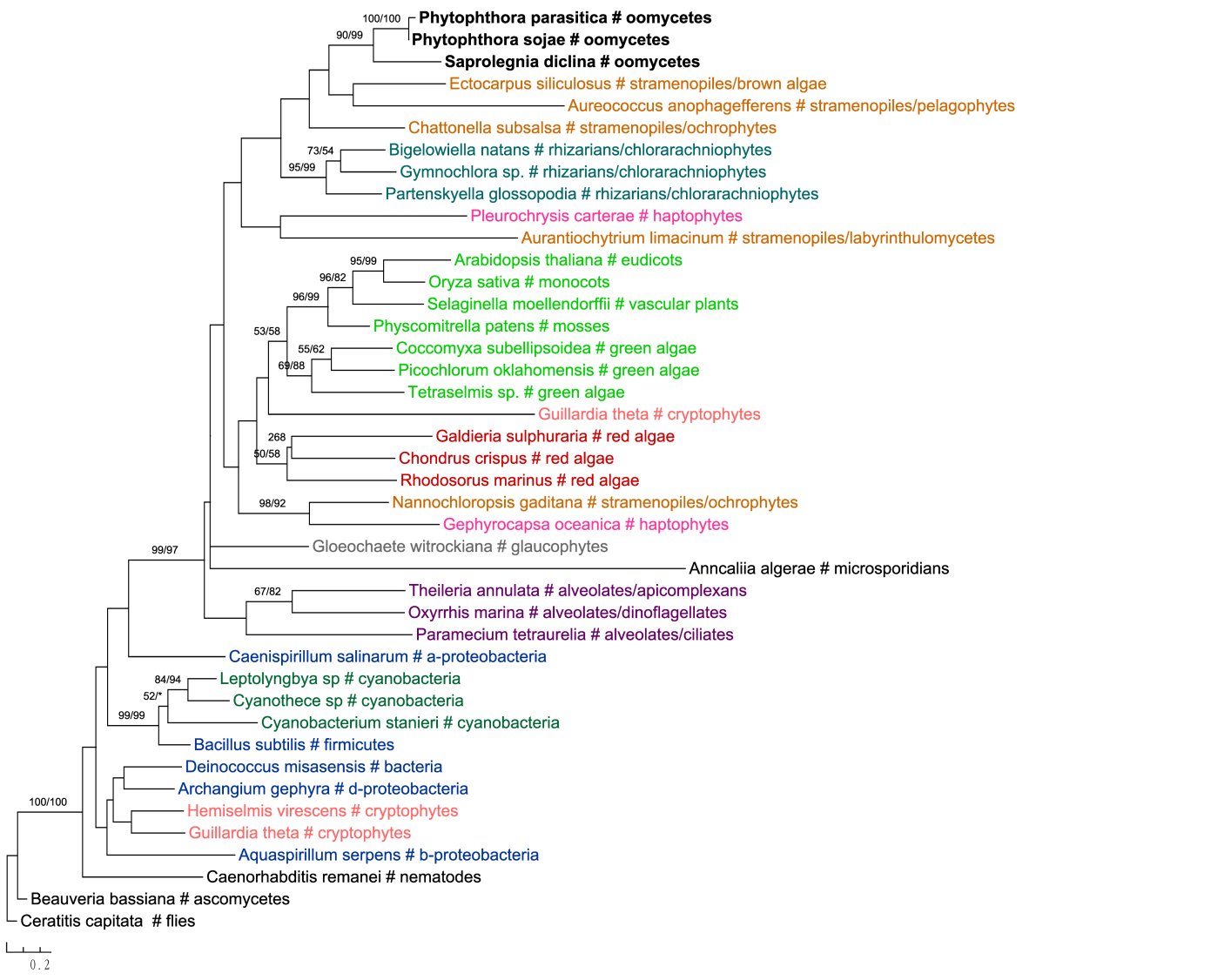


**Supplementary Figure 21.** Molecular phylogeny of tRNA dihydrouridine synthase. Numbers above branches show bootstrap values in percentage for maximum likelihood and distance analyses, respectively. Values below 50% are indicated by asterisks.


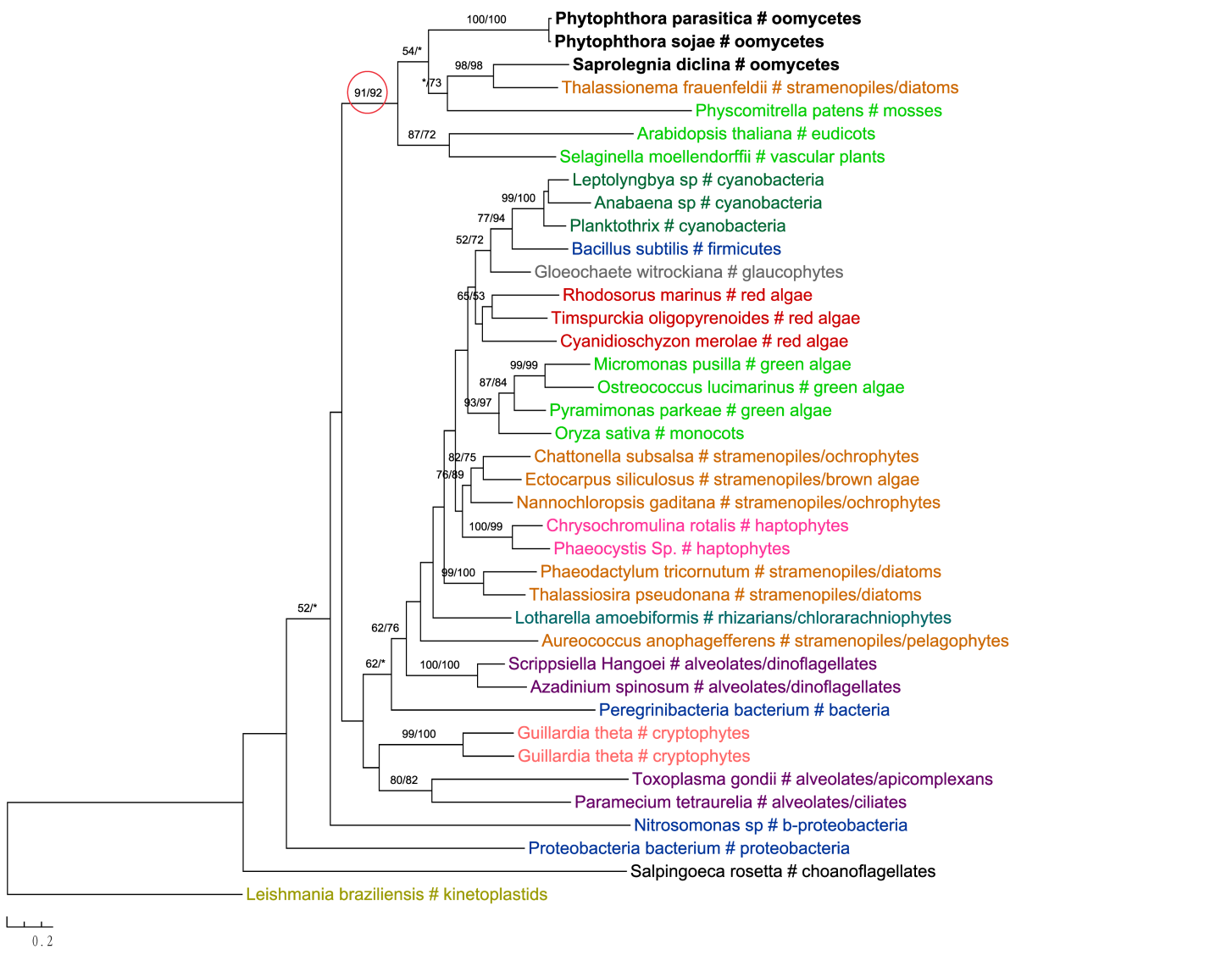


**Supplementary Figure 22.** Molecular phylogeny of probable folate-biopterin transporter. Numbers above branches show bootstrap values in percentage for maximum likelihood and distance analyses, respectively. Values below 50% are indicated by asterisks.


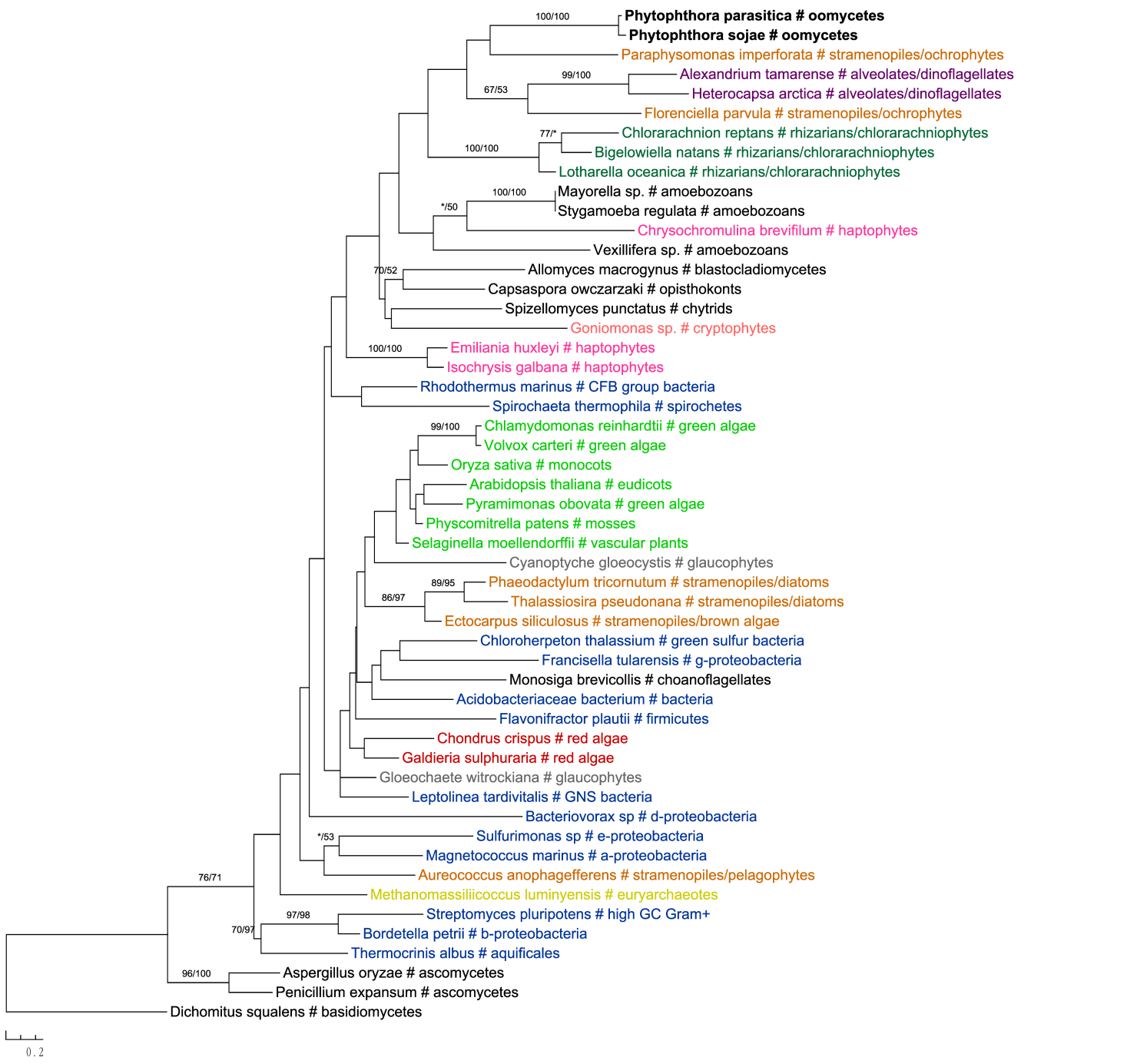


**Supplementary Figure 23.** Molecular phylogeny of prephenate dehydratase family. Numbers above branches show bootstrap values in percentage for maximum likelihood and distance analyses, respectively. Values below 50% are indicated by asterisks.

**Supplementary Table**

**Supplementary Table 1** Additional sequences used for internal customized database construction.

| Species | Taxonomic group | Data type |
| --- | --- | --- |
| *Acanthamoeba castellanii* | Amoebozoa | EST |
| *Acetabularia acetabulum* | Viridiplantae | EST |
| *Alexandrium* *tamarense* | Alveolata | Genome |
| *Allomyces* *macrogynus* | Fungi | EST |
| *Amphidinium carterae* | Alveolata | Genome |
| *Antonospora locustae* | Fungi | EST |
| *Astasia* *longa* | Fungi | EST |
| *Aureococcus anophagefferens* | Stramenopiles | Genome |
| *Bigelowiella natans* | Rhizaria | Genome |
| *Brachionus plicatilis* | Metazoa | Genome |
| *Capitella teleta* | Metazoa | Genome |
| *Chlamydomonas reinhardtii* | Viridiplantae | Genome |
| *Chlorella vulgaris* | Viridiplantae | Genome |
| *Cyanidioschyzon merolae* | Rhodophyta | EST |
| *Cyanophora paradoxa* | Glaucophytes | EST |
| *Diplonema papillatum* | Diplonemida | Genome |
| *Ectocarpus siliculosus* | Stramenopiles | Genome |
| *Emiliania huxleyi* | Haptophyceae | Genome |
| *Euglena gracilis* | Euglenozoa | EST |
| *Galdieria phlegrea* | Rhodophyta | Genome |
| *Glaucocystis nostochinearum* | Glaucocystophyceae | Genome |
| *Guillardia theta* | Cryptophyta | EST |
| *Hartmannella vermiformis* | Amoebozoa | EST |
| *Heterocapsa triquetra* | Alveolata | EST |
| *Histiona aroides* | Jakobida | EST |
| *Hyperamoeba dachnya* | Amoebozoa | EST |
| *Isochrysis galbana_CCMP_1323* | Haptophyceae | EST |
| *Jakoba bahamiensis* | Excavata | EST |
| *Jakoba libera* | Excavata | EST |
| *Karenia* *brevis* | Alveolata | EST |
| *Karlodinium* *micrum* | Alveolata | Genome |
| *Klebsormidium flaccidum* | Viridiplantae | Genome |
| *Lottia gigantea* | Metazoa | EST |
| *Malawimonas californiana* | Excavata | EST |
| *Marchantia polymorpha* | Viridiplantae | Genome |
| *Mastigamoeba balamuthi* | Amoebozoa | EST |
| *Mesostigma* *viride* | Viridiplantae | Genome |
| *Mortierella verticillata* | Fungi | EST |
| *Neocallimastix patriciarum* | Fungi | EST |
| *Nephroselmis olivacea* | Viridiplantae | EST |
| *Nuclearia simplex_strain_2* | Opisthokonta | EST |
| *Ostreococcus tauri* | Viridiplantae | Genome |
| *Oxytricha trifallax* | Alveolata | EST |
| *Paracercomonas marina* | Rhizaria | EST |
| *Pavlova lutheri* | Haptophyceae | EST |
| *Phaeodactylum tricornutum* | Stramenopiles | Genome |
| *Physarum polycephalum* | Amoebozoa | Genome |
| *Phytophthora ramorum* | Stramenopiles | Genome |
| *Polysphondylium* *pallidum* | Amoebozoa | EST |
| *Polytomella parva* | Viridiplantae | EST |
| *Porphyridium purpureum* | Rhodophyta | Genome |
| *Proterospongia choanojuncta* | Choanoflagellida | EST |
| *Prototheca wickerhamii* | Viridiplantae | EST |
| *Pyropia yezoensis* | Rhodophyta | Genome |
| *Reclinomonas americana* | Jakobida | Genome |
| *Rhizopus oryzae* | Fungi | EST |
| *Saccharina japonica* | Stramenopiles | Genome |
| *Saitoella complicata* | Fungi | Genome |
| *Sawyeria marylandensis* | Heterolobosea | EST |
| *Scenedesmus obliquus* | Viridiplantae | EST |
| *Seculamonas ecuadoriensis* | Jakobida | Genome |
| *Sphaeroforma arctica* | Opisthokonta | EST |
| *Sphagnum fallax* | Viridiplantae | Genome |
| *Spironucleus vortens* | Fornicata | Genome |
| *Spizellomyces punctatus* | Fungi | EST |
| *Stachyamoeba lipophora* | Heterolobosea | EST |
| *Streblomastix strix* | Oxymonadida | EST |
| *Taphrina deformans* | Fungi | EST |
| *Tetrahymena thermophila* | Alveolata | Genome |
| *Thecamonas trahens* | Apusozoa | EST |
